# Comparative Analysis of TGF-β/Smad Signaling Dependent Cytostasis in Human Hepatocellular Carcinoma Cell Lines

**DOI:** 10.1371/journal.pone.0072252

**Published:** 2013-08-22

**Authors:** Johanna Dzieran, Jasmin Fabian, Teng Feng, Cédric Coulouarn, Iryna Ilkavets, Anastasia Kyselova, Kai Breuhahn, Steven Dooley, Nadja M. Meindl-Beinker

**Affiliations:** 1 Molecular Hepatology – Alcohol Associated Diseases, Department of Medicine II, Medical Faculty Mannheim, Heidelberg University, Mannheim, Germany; 2 Institute of Pathology, University Hospital Heidelberg, Heidelberg, Germany; 3 Institut National de la Sante et de la recherche Medicale UMR991, University of Rennes, Pontchaillou University Hospital, Rennes, France; National Cancer Center, Japan

## Abstract

Hepatocellular carcinoma (HCC) is a major public health problem due to increased incidence, late diagnosis and limited treatment options. TGF-β is known to provide cytostatic signals during early stages of liver damage and regeneration, but exerts tumor promoting effects in onset and progression of liver cancer. To understand the mechanistic background of such a switch, we systematically correlated loss of cytostatic TGF-β effects with strength and dynamics of its downstream signaling in 10 HCC cell lines. We demonstrate that TGF-β inhibits proliferation and induces apoptosis in cell lines with low endogenous levels of TGF-β and Smad7 and strong transcriptional Smad3 activity (PLC/PRF/5, HepG2, Hep3B, HuH7), previously characterized to express early TGF-β signatures correlated with better outcome in HCC patients. TGF-β dependent cytostasis is blunted in another group of cell lines (HLE, HLF, FLC-4) expressing high amounts of TGF-β and Smad7 and showing significantly reduced Smad3 signaling. Of those, HLE and HLF exhibit late TGF-β signatures, which is associated with bad prognosis in HCC patients. RNAi with Smad3 blunted cytostatic effects in PLC/PRF/5, Hep3B and HuH7. HCC-M and HCC-T represent a third group of cell lines lacking cytostatic TGF-β signaling despite strong and prolonged Smad3 phosphorylation and low Smad7 and TGF-β expression. Inhibitory linker phosphorylation, as in HCC-T, may disrupt C-terminally phosphorylated Smad3 function. In summary, we assort 10 HCC cell lines in at least two clusters with respect to TGF-β sensitivity. Cell lines responsive to the TGF-β cytostatic program, which recapitulate early stage of liver carcinogenesis exhibit transcriptional Smad3 activity. Those with disturbed TGF-β/Smad3 signaling are insensitive to TGF-β dependent cytostasis and might represent late stage of the disease. Regulation of this switch remains complex and cell line specific. These features may be relevant to discriminate stage dependent TGF-β functions for the design of efficient TGF-β directed therapy in liver cancer.

## Introduction

HCC is the third most lethal cancer in the world with dramatically rising incidence [1,2]. Limited treatment options and delayed diagnosis caused by late occurring symptoms [3] highlight the urgent need to characterize the heterogeneity of oncogenic mechanisms in HCC and to identify early disease biomarkers and new drugable targets.

TGF-β, a multifunctional cytokine, signals via canonical Smad dependent and non-canonical Smad independent pathways regulating the expression of more than 500 genes. Canonically, TGF-β binds to TGF-β receptors type II (TβRII) which subsequently recruit and activate the type I receptors TβRI/ALK-5 by phosphorylation, leading to downstream C-terminal phosphorylation of receptor (R)-Smad proteins. Phospho-activated R-Smads then complex common mediator (Co)-Smad4, translocate into the nucleus and act as transcription factors in concert with co-activators and co-repressors [4].

TGF-β is prominent in damaged liver and represents a key regulator of hepatic stellate cell activation and liver fibrogenesis upon most types of liver damage. It displays cytostatic effects inducing apoptosis in distinct hepatocytes and interfering with hepatocyte proliferation during liver regeneration. Chronic liver damage frequently progresses towards cirrhosis and HCC. During this process, TGF-β is assumed to switch from cytostatic to oncogenic action on hepatocytes becoming a plasticity factor that induces epithelial mesenchymal transition (EMT), cytokine and receptor production, migration and invasion.

Malignant cells can circumvent the cytostatic effects of TGF-β either by mutational inactivation of core pathway components, as TGF-β receptors or Smad proteins, or by interfering with cytostatic branches of TGF-β signaling (for review, 5). Such mutations were described in colorectal, pancreatic, ovarian, gastric, and head and neck carcinomas [5], whereas they are rare in HCC [6]. This indicates that transformed hepatocytes principally retain an intact TGF-β signaling machinery with alterations of the tumor-suppressive arm only. Although missing a general mechanism of this process in HCC, several studies have provided hypotheses. Thus, a link between deletion of the adaptor protein ELF, expression of its inhibitor PRAJA and defective Smad3 signaling leading to significant liver disease was reported [7,8]. Further, constitutively activated Ras was shown to act as initiating step switching TGF-β effects from cytostatic to tumorigenic [9]. TGF-β inhibitory Smad7 was found upregulated in a limited number of investigated patients [10,11]. Then JNK mediated linker phosphorylation of Smad3 inactivated cytostatic Smad3 signaling and facilitated hepatocarciongenesis [12]. Although such dual role of TGF-β has long been noted and potential routes for a tumorigenic switch were described, robust mechanistic markers to sub classify patient cohorts are still not available. This is, however, of utmost relevance since TGF-β directed therapy is currently envisaged and clinical trials are underway for late stage HCC patients.

In order to expand the knowledge, we thoroughly investigated TGF-β signaling and cytostatic effects in 10 HCC cell lines, for the first time under strictly comparable conditions. Our results highlight the heterogeneity of HCC cell lines in response to TGF-β, but allowed identification and characterization of two general groups - one being responsive the other being insensitive to TGF-β-induced cytostatic program (i.e. inhibition of proliferation and/or induction of cell death). While the former expressed low endogenous TGF-β and Smad7 levels and showed significant Smad3 transcriptional activity, the latter exhibited the opposite features. We concluded that although HCC cell lines are generally thought to represent late stages of liver cancer, they display a diverse picture regarding TGF-β signaling. In line with heterogeneity of HCC tumors in patients, the heterogeneity of HCC cell lines obviously reflects different stages and mechanisms of the disease. Thus, our results provide a unique opportunity to select relevant HCC cell lines to investigate specific (especially TGF-β related mechanisms) driving HCC onset and progression.

## Materials and Methods

### LDH Assay

After starvation for 8h, cells were treated with TGF-β for 72h. For HCC-M and HuH7, starvation medium was supplemented with 1% heat inactivated FCS. LDH content in supernatant and adherent cells (disrupted with 1% Triton X-100 in HBSS) was detected using Cytotoxicity Detection Kit (Roche Diagnostics GmbH, Mannheim, Germany). Cell death was calculated as percentage of LDH in the medium as compared to total LDH levels.

### MTT Assay

Cells were cultured in medium containing 0.25% heat inactivated FCS with 5ng/ml TGF-β for 2 or 6 days (addition of new FCS and TGF-β after 3 days). 4h after adding MTT reagent (final concentration 500µg/ml), reduced dye in viable cells was resolved in acidified DMSO solution (10% SDS, 0.6% glacial acid in DMSO). Absorbance was measured at 570nm (reference 630nm). Values of treated samples were normalized to untreated controls.

### RNA Extraction, Reverse Transcription, Real Time PCR Analysis

HCC cell lines were treated with TGF-β for 2 or 24h. RNA was isolated using RNeasy Mini Kit, integrity ensured by agarose gel electrophoreses and reverse transcribed using QuantiTect Reverse Transcription Kit (QIAGEN, Hilden, Germany). mRNA levels of Smad7, TGF-β1, TβRI, TβRII and 18S-rRNA were detected using TaqMan® probes and TaqMan® Universal PCR Master Mix, No AmpErase® UNG; PRAJA, ELF, Bim, PAI-1 and 18S-RNA using Power SYBR® Green PCR Master Mix (Applied Biosystems, Foster City, CA, USA). Primer pairs used for SYBR green Real time PCR were as followed: PRAJA (For: 5’-TCGCCATTTTCCACTACTCGT-3’; Rev: 5’-GTTCCCGAACTCTCGCTGT-3’), ELF (For: 5’-AGCTGGAAGGCAGATTCAAG-3’; Rev 5’-CGTCCATCTCGAAGGTCAGT-3’), Bim (For: 5’-TAAGTTCTGAGTGTGACCGAGA-3’; Rev 5’-GCTCTGTCTGTAGGGAGGTAGG-3’), PAI-1 (For: 5’-CACAAATCAGACGGCAGCACT-3’; Rev 5’-CATCGGGCGTGGTGAACTC-3’), Smad4 (For: 5’-GCTGCTGGAATTGGTGTTGATG-3’; Rev 5’-AGGTGTTTCTTTGATGCTCTGTCT-3’) and 18S rRNA (For: 5’- AAACGGCTACCACATCCAAG-3’; Rev 5’-CCTCCAATGGATCCTCGTTA-3’). Following assays were obtained from Applied Biosystems and used for TaqMan, Real time PCR: Smad (Hs00178696_m1), TGF-β1 (Hs00171257_m1), TGFBR1 (TβRI, Hs00610318_m1), TGFBR2 (TβRII, Hs00559661_m1), Smad2 (Hs00998181_gH), Smad3 (Hs00969205_g1) and 18S rRNA (Hs03003631_g1). Relative expression levels were determined using the ΔΔCt method (reference 18S-rRNA level). As 18S-rRNA expression values are very stable (see Figure S1) only one reference gene was used according to literature [13].

### Immunoblot Analysis

Blotted proteins were detected using antibodies against pSmad2C (Ser465/467), Smad2 (D43B4), Smad3, Akt, pAKT (Ser473) (587F11), pc-JUN (Ser63), pP38 MAPK (Thr180/Tyr182) (12F8), cleaved Caspase-3 (Asp175) (5A1E), PARP, BCL-2 (50E3) and BCL-XL (54H6) (Cell Signaling Technology, Danvers, MA, USA), Smad4 (B-8), PCNA (F-2), c-MYC (2Q329), ERK1/2 (MK1) and pERK (E-4) (Santa Cruz Biotechology, Santa Cruz, California, USA.), P21WAF1/Cip1 (CP74) (Sigma Aldrich, St. Louis, Missouri, USA) and pSmad3L (pS423/425) (Epitomics, Burlingame, California, USA). Secondary antibodies were goat anti-rabbit IgG-HRP or goat anti-mouse IgG-HRP (Santa Cruz Biotechnology, Santa Cruz, California, USA). Antibodies’ sources, dilutions and buffer conditions are given in table S1. All experiments were performed multiple times, number of independent experiments and densitometric analysis are given in figures S2, S3, S4, S5 and S6.

### Immunofluorescent Staining of Linker Phosphorylated Smad3

Cells were seeded with a density of 300.000 cells per well in 6-well plates without further treatment. After 1 day, cells were washed twice with PBS pH7.4, fixed in 4% PFA/PBS for 10 minutes, permeabilized with 0.3% Triton X-100 v/v in PBS pH 7.4 for 5 min, fixed a second time with 2% PFA/PBS for 5 min and washed 3 times in PBS. Fixed and permeabilized cells were blocked in 1% (w/v) BSA/PBS pH 7.4 for 1h, then incubated with rabbit-anti Smad3L antibody (Immunobiological Laboratories No. 28029) over night at 4°C, washed 3 times in PBS and incubated with Alexa-555-goat anti–rabbit IgG (Molecular Probes/Invitrogen) for 50 min at room temperature. Nuclei were stained with DRAQ5 (Biostatus Ltd, Shepshed Leicestershire, UK) in PBS for 15 min. Fixed samples were washed 3 times with PBS pH 7.4 and mounted on glass slides using DakoCytomation Fluorescent Mounting Medium (DakoCytomation, Hamburg, Germany). Confocal images were obtained with a Leica laser scanning spectral confocal microscope, model DM IRE2, with an HCX PL Apo 40x/1.32 numeric aperture oil objective (Leica Microsystems, Wetzlar, Germany). Excitation was performed with an argon laser emitting at 488 nm, a krypton laser emitting at 568 nm, and a helium/neon laser emitting at 633 nm. Images were acquired with a TCS SP2 scanner and Leica Confocal software, version 2.5 (Leica Microsystems).

### Cell Culture

HCC-M, HCC-T, HepG2, Hep3B, HuH7, PLC/PRF/5 (PLC, Alexander), HLE, HLF, FLC-4 and HuH6 cells were cultured in DMEM (Lonza Group Ltd., Cologne, Germany) supplemented with 2mM glutamine (PAA Laboratories GmbH, Cölbe, Germany) and 10% FCS (Invitrogen, Darmstadt, Germany) at 37°C and 5% CO_2_ in a humidified incubator. Experiments were conducted between passage numbers 2 and 12 without full confluency throughout experiments. Absence of mycoplasma contamination was confirmed by Venor®GeMtest (Minerva Biolabs GmbH, Berlin, Germany). Prior to experiments, medium was changed to starvation medium without FCS for at least 7h, if not indicated otherwise. 5ng/ml TGF-β (PeproTech GmbH, Hamburg, Germany) were used. Cell line characteristics are given in table S2 in the supplementary data.

### Smad3 Transcriptional Activity

Adherent cells were infected for 2h with adenovirus carrying a Smad3 reporter construct, (CAGA) _9_MLP-Luc [14] or control adenovirus with β-Galactosidase [15]. After overnight starvation, TGF-β was added for 9h. Luciferase and β-Galactosidase activities were analyzed using Luciferase Assay Reagent and β-Galactosidase Enzyme Assay (Promega, Madison, WI, USA). After normalizing luciferase activity to β-Gal expression, TGF-β dependent induction of Smad3 transcriptional activity was normalized to the untreated control sample. Each condition was analyzed in triplicates.

### Smad2 transcriptional activity

Smad2 transcription factor complexes recognize activin response elements (ARE). Smad2 is unable to bind DNA and needs assistance by, e.g. Fast-1, as cofactor. Hence, a luciferase gene under the control of ARE was co-transfected with a Fast-1 expression plasmid to evaluate Smad2/Smad4 transcriptional activity. For this, HCC cell lines were cultured at a confluency of 70-80%. ARE-Luc, Fast-1 and a β-Galactosidase control vector (pCR3lacZ; Invitrogen) at a ratio of 6:2:1 were introduced into the cell using Lipofectamine 2000 (Invitrogen, Darmstadt, Germany) according to the manufacturer’s protocol. After transfection, cells were starved over night until treatment with TGF-β for 9h. β-Galactosidase and Luciferase assay was performed as described above.

### Smad7 Promoter Activity

β-Galactosidase control vector (pCR3lacZ; Invitrogen) and Smad7 promoter deletion mutant, p(-625 SacI)-Smad7prom-Luc, constructed from the 1,321-bp rat Smad7 promoter region (-1276 to -41) [16] were transiently transfected using Lipofectamine 2000 (Invitrogen, Darmstadt, Germany). Immediately post-transfection, cells were starved for 24h until treatment with TGF-β for 6h. β-Galactosidase and Luciferase assays were performed as described above.

### Knockdown of Smad2 and Smad3 with siRNA

Hep3B, HuH7 and PLC/PRF/5 cells were cultured at medium density to perform siRNA knockdown experiments. Smad2 and Smad3 or unspecific siRNA (Order No SI02757496, SI00082495 and 1027281; Qiagen, Hilden, Germany) was introduced into the cells using Lipofectamine RNAiMax (Invitrogen, Darmstadt, Germany) according to the manufacturer’s protocol but with 2 µl RNAiMax per ml medium. Final siRNA concentrations were 10 µM for Hep3B and PLC/PRF/5 and 20 µM for HuH7 cells. Knockdown was allowed to establish for 48 h in medium supplemented with 1% heat inactivated FCS. Cells were treated with starvation medium supplemented with 5 ng/ml TGF-β for 1 h (proof of knockdown; Western blot analysis) or 72 h (LDH assay). Medium for HuH7 cells additionally contained 1% heat inactivated FCS. LDH and Western blot analysis was performed as described above. Additionally, content of LDH in adherent cells was used to evaluate proliferation.

### Clustering Analysis and Statistics

Clustering analysis was performed by using Cluster 3.0 software and the data were further visualized with TreeView 1.6 [17]. Results are shown as means +/- standard error of 3-5 (MTT Assay), 3-4 (LDH Assay), 3-4 (Real Time PCR, basal expression), 2-3 (Real Time PCR, induced expression), 3 (Smad3 transcriptional und Smad7 promotor activity), 2-3 (Smad2 transcriptional activity) independent experiments. Significant differences were determined by two-tailed unpaired Student’s t test.

## Results

### Cytostatic TGF-β response is maintained in Hep3B, HuH7 and PLC HCC cell lines

**Figure 1:**
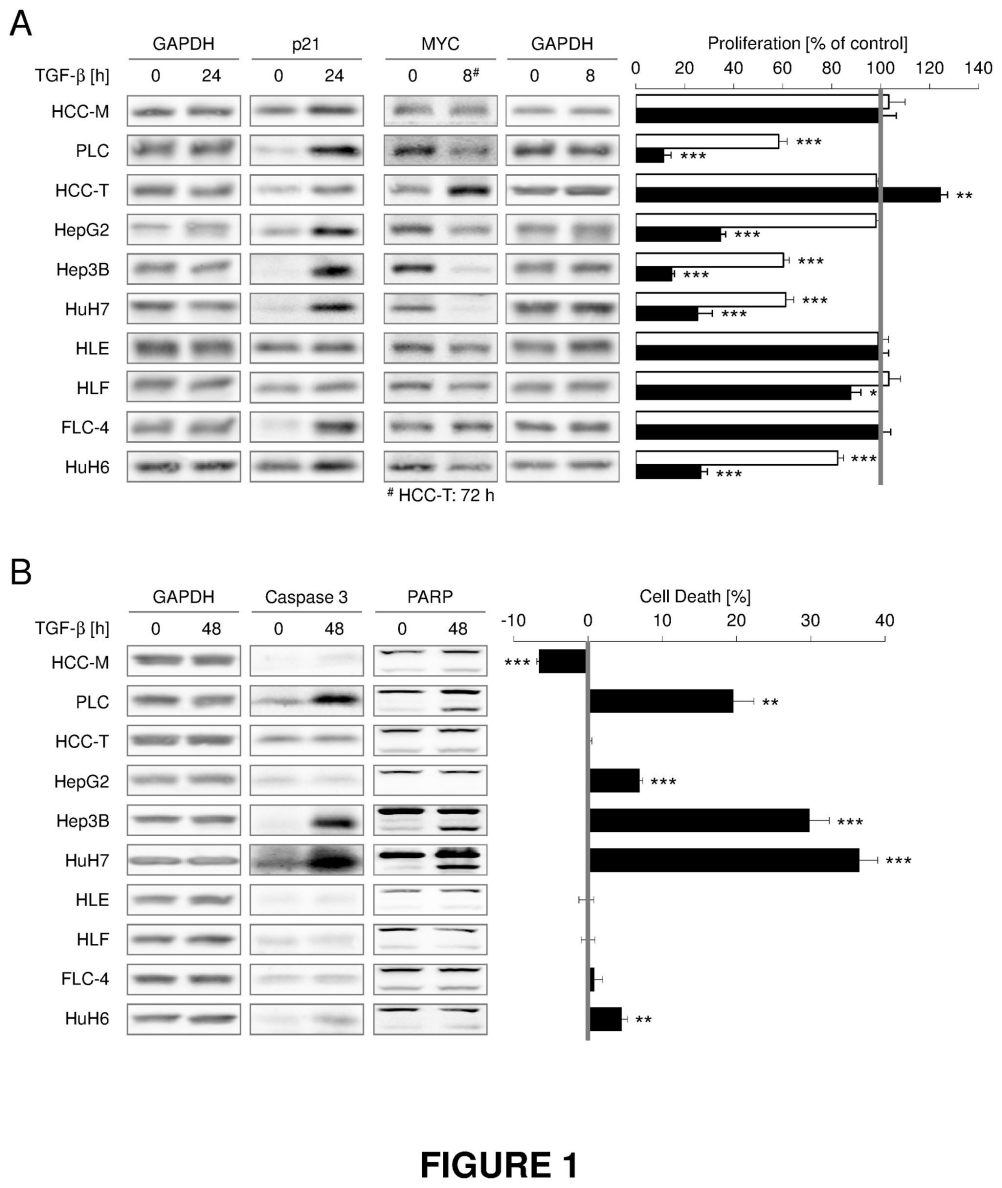
TGF-β induces cell death and/or inhibits proliferation in PLC, HepG2, HuH6, Hep3B and HuH7. (A) Left side: HCC cell lines were treated with 5 ng/ml TGF-β for indicated time points. Changes in c-MYC and P21 expression were detected using Western blot analysis with GAPDH as loading control. As HCC-T cells show a delayed TGF-β response, c-MMYC was induced after 72 h of TGF-β treatment. Right side: After TGF-β treatment for 0 (grey line), 2 (white bars) or 6 days (black bars) cell proliferation was evaluated by MTT assays. Treated samples were normalized to the corresponding control. (B) Left side: Western blot analysis of PARP and Caspase 3 cleavage using GAPDH expression as loading control after 0 and 48 h TGF-β treatment. In the case of HuH7 cells, the control sample was treated with TGF-β for 3 h. Right side: Cell death induced by 5 ng/ml TGF-β over 72 h (filled bars) was quantified by detecting LDH release normalized to total amount of LDH. Untreated samples were defined as 0 (grey line). Significant differences are indicated as * p < 0.05, ** p < 0.01 and *** p < 0.001 (Student’s t test).

Sensitivity to TGF-β induced cytostasis was evaluated in 10 HCC cell lines. 2 days TGF-β treatment significantly inhibited cell proliferation in Hep3B, HuH7 and PLC, as determined by MTT-assay (Figure 1A, right panel), HepG2 and HuH6 displayed no or a weak response after 2 days, respectively. However, they show a strong response 6 days upon TGF-β addition. Proliferation of HCC-M, HCC-T, HLE, FLC-4 and HLF was not reduced. HCC-T even displayed an increased proliferation after 6 days treatment. In line with MTT-assay data, expression of proliferation associated marker P21 showed the strongest induction comparing before and after TGF-β treatment in those cell lines with TGF-β dependent proliferation inhibition. Consistently, expression of proliferation facilitating protein c-MYC was specifically down regulated in cell lines sensitive to TGF-β induced proliferation control, whereas it was even induced late in HCC-T cells (Figure 1A, left panel, Figure S2). Western Blot analysis was not sensitive enough to confirm weak cell death (+7%) of HepG2 upon TGF-β treatment on protein level.

Besides controlling proliferation, TGF-β is a prominent modulator of hepatocyte apoptosis. We analyzed apoptosis of TGF-β treated cells by LDH release to the medium and cleavage of PARP and Caspase3 (Figure 1B, Figure S2). Again, Hep3B, HuH7 and PLC cells showed increased cell death after 72h TGF-β stimulation, whereas HepG2 and HuH6 exhibited very low TGF-β induced cell death rates. Increased cytotoxicity was accompanied by elevated levels of PARP and Caspase3 cleavage (Figure 1B). No significant pro apoptotic effect of TGF-β was found in the other cell lines. Our results revealed heterogeneous cytostatic TGF-β effects in HCC cell lines. Sensitivity for cell death induction and proliferation inhibition generally occur in common (Hep3B, HuH7, PLC; to a lower extent HepG2, HuH6). HCC-M, HCC-T, HLE, HLF and FLC-4 are completely resistant to cytostatic TGF-β effects.

### Upregulated TGF-β production and high expression of inhibitory Smad7 correlate with loss of cytostatic response in HCC cell lines

**Figure 2:**
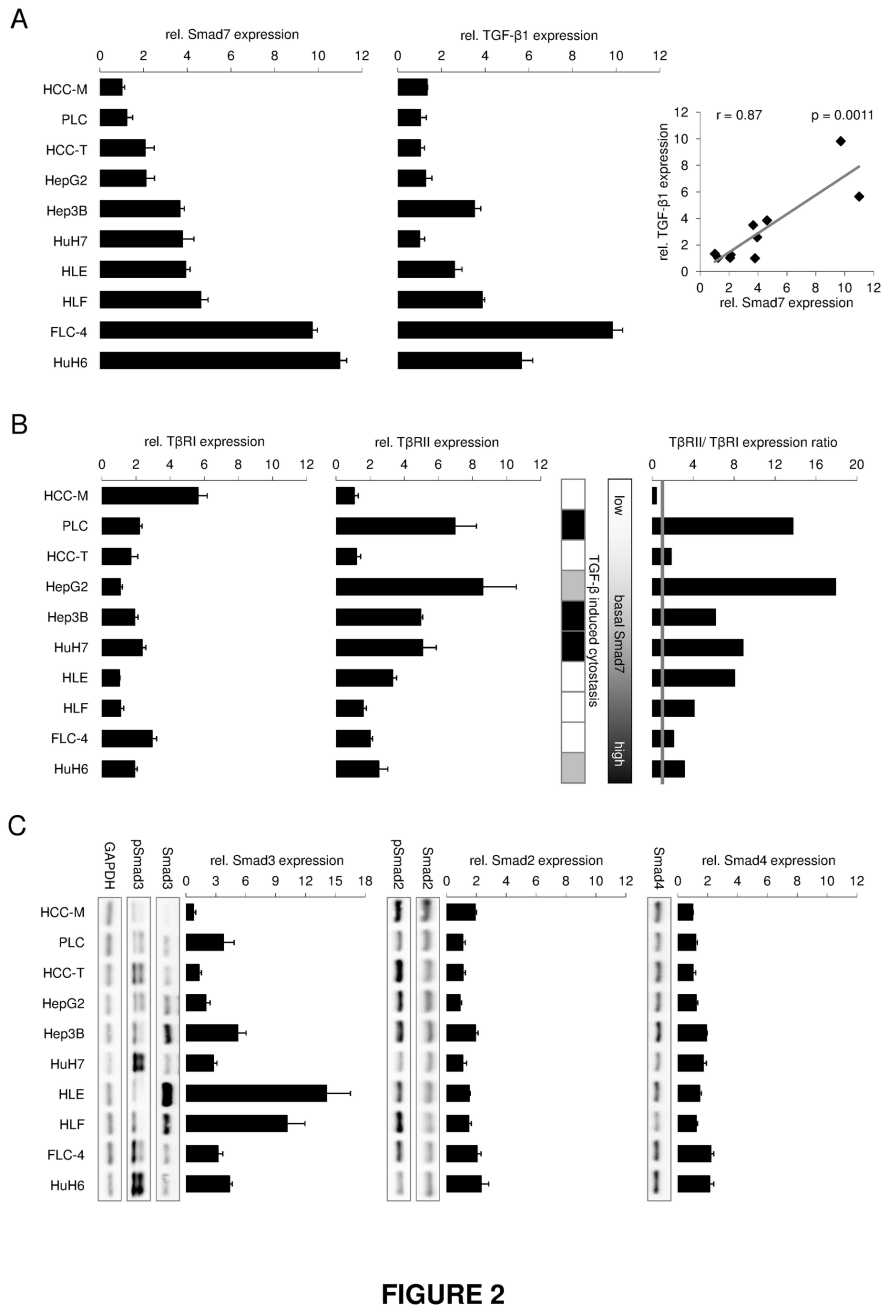
Expression levels of TGF-β/Smad signaling components in HCC cell lines. Relative TGF-β1 and Smad7 (A), TGF-β receptor I (TβRI) and II (TβRII) (B) and Smad2, Smad3, and Smad4 (C) expression levels were detected using real time PCR. Expression of 18s rRNA was used as reference and results were analyzed with the ΔΔCt method. A strong correlation of Smad7 and TGF-β1 expression was identified by calculating the Pearson coefficient (r = 0.87; p = 0.0011) (A). Further, TβRII expression (filled bars) was generally increased compared to the expression of TβRI (grey line) in the same cell line (B, right). Additionally, total and phosphorylated Smad2 and Smad3 as well as basal Smad4 protein levels were evaluated by Western Blot using GAPDH as loading control (C).

We next tried to correlate (loss of) cytostatic response with disposability of TGF-β signaling components and dynamics of its downstream signaling. One feature of progressed cancer cells is TGF-β production and autocrine stimulation. Therefore, we analyzed endogenous TGF-β expression by qRT-PCR. A broad spectrum of expression levels was found in the cell lines (1-10 fold) (Figure 2A). Interestingly, intrinsic levels of inhibitory Smad7 similarly varied and relative expression strongly correlated with that of TGF-β1 (Pearson correlation, r = 0.87, p = 0.0011) (Figure 2A) increasing in the following order: HCC-M, PLC, HCC-T, HepG2, Hep3B, HuH7, HLE, HLF, FLC-4 and HuH6. Cytostatic TGF-β effects could be correlated to low endogenous TGF-β/Smad7 expression levels (HCC-M, HCC-T, PLC, HepG2, HuH7, exception Hep3B). The special position taken by HCC-M and HCC-T has to be pointed out. Both cell lines express rather low TGF-β and Smad7 levels, but do not respond to TGF-β mediated cytostasis. This issue will be discussed further below.

Endogenous levels of TGF-β receptor I (TβRI, ALK-5) were relatively even within the different cell lines, although HCC-M displayed especially high levels (Figure 2B). TGF-β receptor II (TβRII) expression was upregulated in cell lines with an asserved cytostatic TGF-β response, showing high (HepG2 and PLC) or medium (Hep3B and HuH7) TβRII mRNA levels (Figure 2B). HuH6 with delayed proliferation inhibition upon TGF-β treatment expressed low amounts of TβRII, whereas HLE cells lacking TGF-β dependent cytostasis displayed intermediate expression levels.

Receptor-Smad2 and co-Smad4, like TβRI, were equally expressed in the different HCC cell lines, whereas Smad3 exhibited strikingly high mRNA and protein expression in HLE and HLF (Figure 2C, Figure S3). However, no significant correlation between Smad3 expression levels and the HCC cells cytostatic response could be concluded.

### TGF-β effects on expression of its signaling components in HCC cell lines

**Figure 3:**
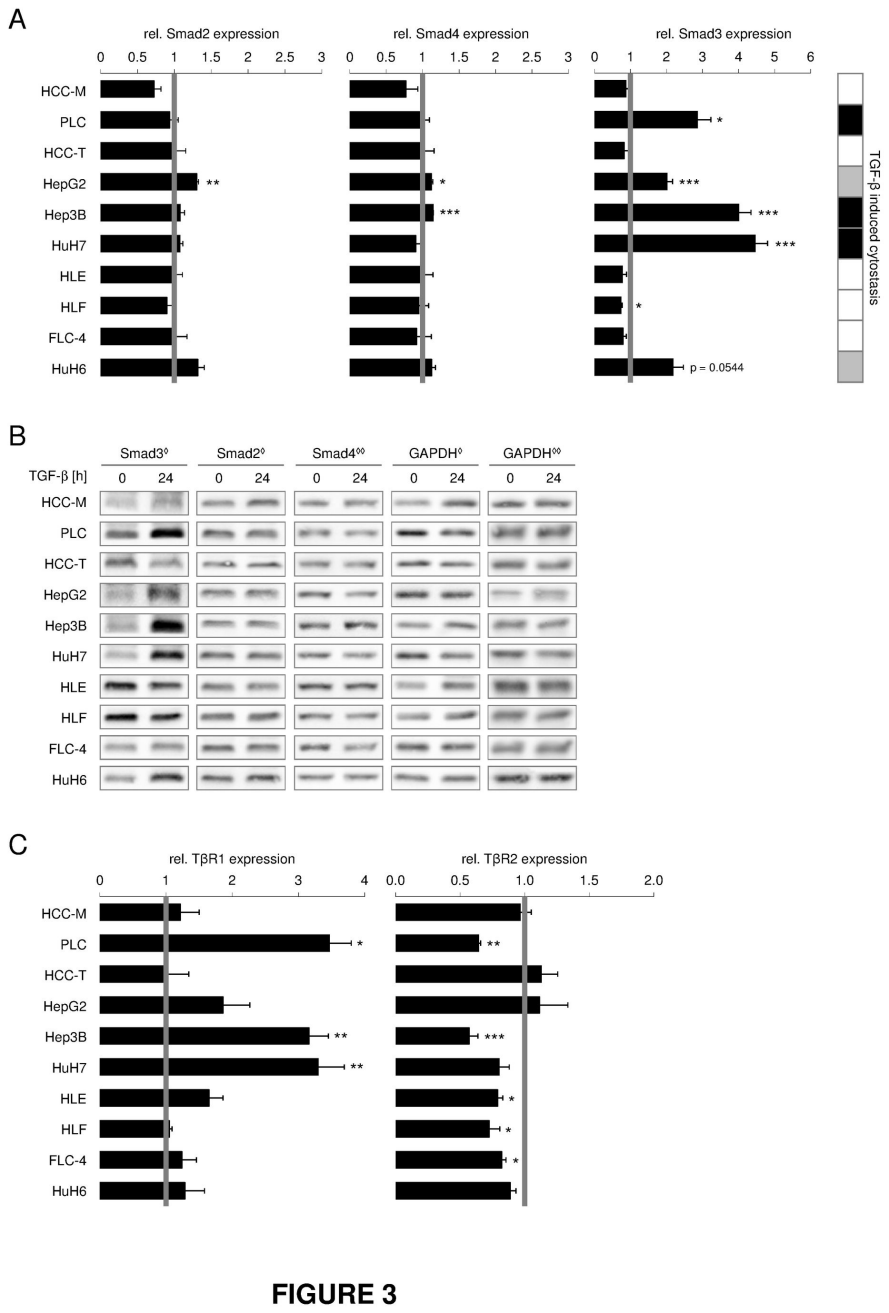
Induction of Smad3 and TβRI expression correlates with cytostatic responsiveness upon TGF-β treatment. Cells were cultured with or without 5 ng/ml TGF-β for 24 h. Changes in Smad2, Smad3 and Smad4 levels after TGF-β treatment were evaluated by (A) Real Time PCR and (B) Western Blot analysis using 18S rRNA or GAPDH as reference genes. ^◊^ and ^**◊ ◊**^ indicate which GAPDH belongs to Smad2 and Smad3 or Smad4, respectively. (C) TGF-β dependent expression levels of TGF-β Receptor I (TβRI) and II (TβRII) were detected and correlated against untreated samples using real time PCR with 18S rRNA as reference gene. Untreated samples are shown as grey lines, while filled bars display TGF-β treated samples. Significant differences are indicated as * p < 0.05, ** p < 0.01 and *** p < 0.001 (Student’s t test).

**Figure 4:**
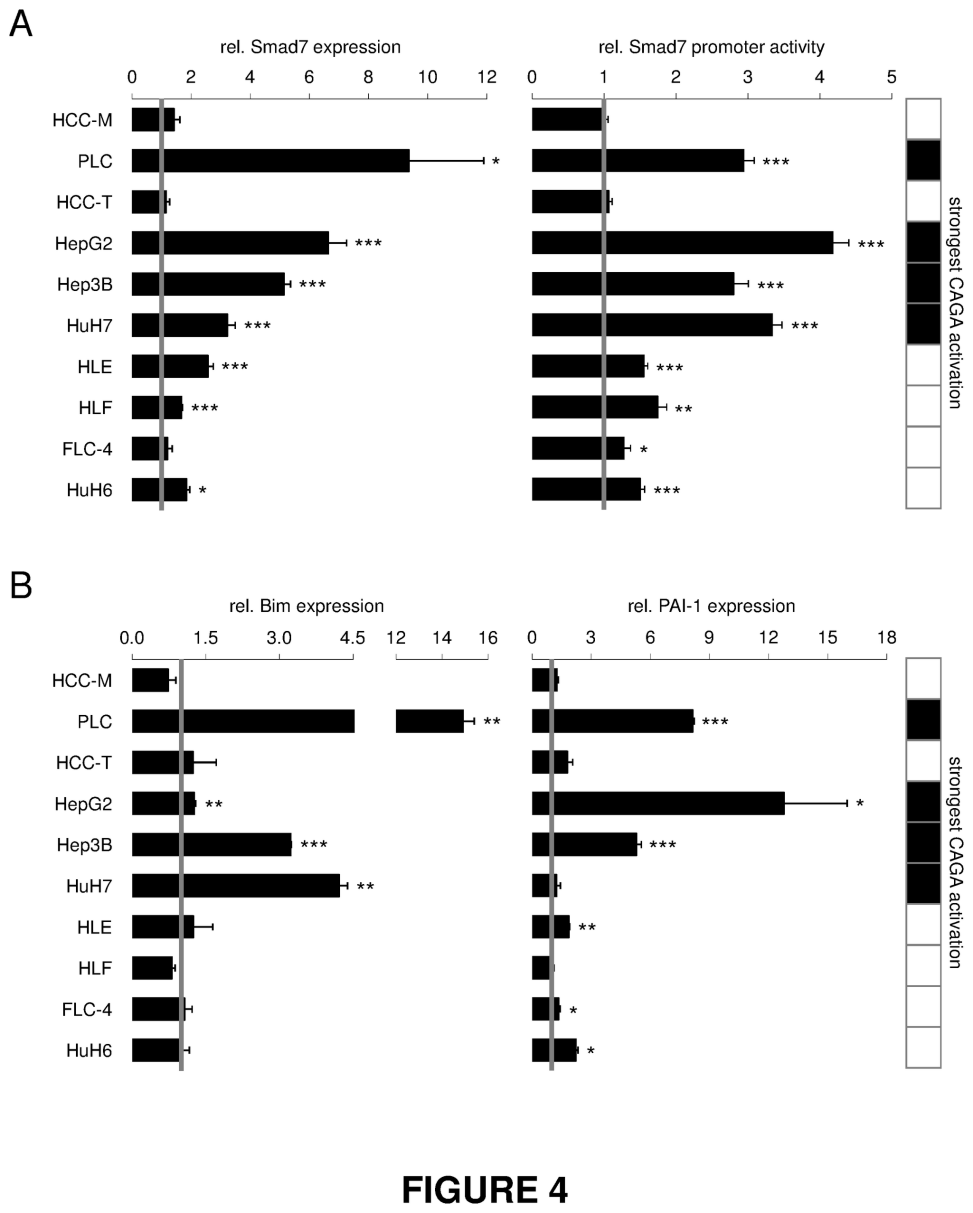
Induction of Smad7 expression by TGF-β correlates with cytostatic responsiveness. (A) Left side: Cells were treated with or without TGF-β for 2 h. Smad7 expression and 18S rRNA levels as reference gene were detected by real time PCR. Right side: For evaluation of transcriptional activity, HCC cell lines were transfected with a construct containing a luciferase gene under control of the Smad7-promotor and treated with TGF-β for 6 h. Treated samples were correlated to untreated controls. (B) Changes in expression levels of Smad3 target genes Bim and PAI-1 were detected after 2 h (PAI-1) or 24 h (Bim) using real time PCR analysis with rS18 as reference gene. The tables highlight cell lines with highest CAGA activity (black fields). Significant differences are indicated as * p < 0.05, ** p < 0.01 and *** p < 0.001 (Student’s t test).

In order to mimic the response of hepatocytes to TGF-β secreted by other cell types, we investigated the impact of TGF-β stimulation on expression of TGF-β signaling components. Smad2 and Smad4 (Figure 3A, 3B, Figure S4) levels did not vary upon 24h TGF-β treatment, whereas Smad3 (Figure 3A, 3B, Figure S4) and Smad7 (Figure 4A) expression was significantly induced mostly in cytostasis responsive cell lines (including HuH6), 24h and 2h after TGF-β treatment. TGF-β-induced expression of Smad7 (Figure 4A) was inversely correlated with intrinsic Smad7 expression, excluding HCC-M and HCC-T (Figure 2.A).

While TβRII levels did not vary upon TGF-β stimulation, TβRI expression was strongly induced in cytostatic responsive cell lines (PLC, Hep3B, and HuH7) (Figure 3C), suggesting a regulatory role for TβRI in driving the effects of TGF-β dependent cytostasis.

### Prolonged vs short term Smad2 signaling in HCC cell lines

**Figure 5:**
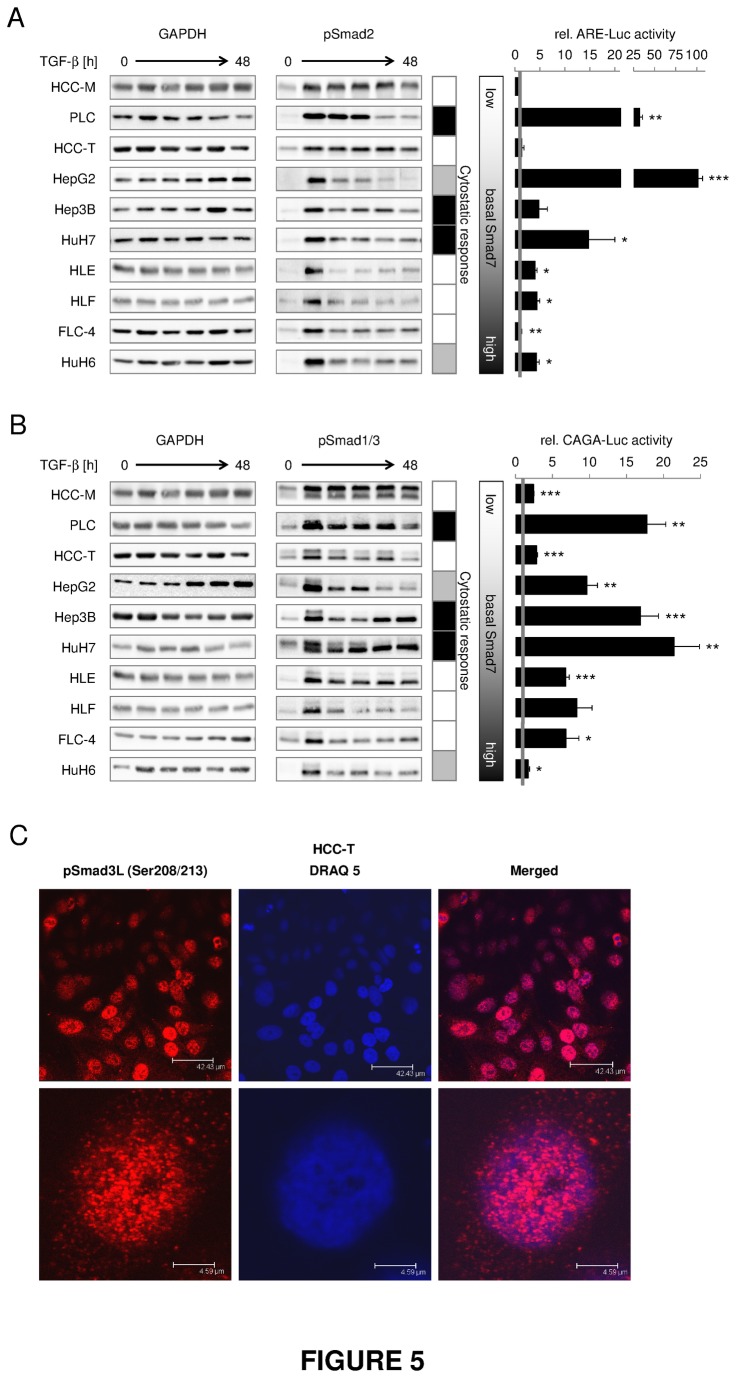
While Smad2 phosphorylation duration correlates to TGF-β and Smad7 expression, cytostatic responsiveness relates to CAGA-reporter-activation. HCC cell lines were treated with 5 ng/ml TGF-β for 0, 1 (3),, 7, 24 and 48 h. (A) Left side: Western blot analysis of Smad2 phosphorylation and GAPDH expression. Right side: To evaluate TGF-β dependent transcriptional activity of Smad2, cells were transfected with plasmids carrying a luciferase gene under control of the activin response element (ARE) and additionally with a FAST-1 expression construct. Luciferase activity of cells treated with 5 ng/ml TGF-β for 9 h was correlated to untreated control samples. (B) Left side: Western blot analysis of Smad1/3 phosphorylation (1: upper/3: lower band); loading control GAPDH. Right side: TGF-β dependent transcriptional activity of Smad3 was evaluated 9 h after TGF-β treatment in cells infected with an adenovirus carrying a luciferase gene controlled by a CAGA response element. Treated samples were correlated to the corresponding untreated control. In the table, cell lines marked as black, show TGF-β induced cell death and growth inhibition whereas cells marked as grey mainly react with the latter one. The gradient from white to black displays increasing basal Smad7 expression levels. (C) Immunofluorescent detection of linker phosphorylated Smad3 in uninduced HCC-T cells. Red fluorescence indicates nuclear localization of pSmad3L. Blue staining indicates nuclei stained with DRAQ5. Significant differences are indicated as * p < 0.05, ** p < 0.01 and *** p < 0.001 (Student’s t test).

As altered expression levels of signaling components may not necessarily reflect activated signal transduction, we investigated the phosphorylation i.e. activation of Smad2 (Figure 5A, Figure S5). Some cell lines (HCC-M, HCC-T, PLC, Hep3B, HuH7) exhibited a prolonged pSmad2 signal after continuous stimulation with TGF-β up to 48h, whereas others (HepG2, HLE, HLF) faded out after 1-7h of TGF-β treatment. In FLC-4 und HuH6 cells pSmad2 signal peaks after 1 hour and then stabilizes on a lower level. This let us sort the cell lines into transiently and long term activated responders with regard to Smad2 phosphorylation.

Smad7 is a potent inhibitor of TGF-β signaling. In line, our data suggest that Smad7 expression is critical in controlling the duration of Smad2 activation. We found a negative correlation (Pearson coefficient r=-0.54) between endogenous (not induced) Smad7 expression levels and the stability of TGF-β induced pSmad2 signals (Figure 2A, Figure 5A). Except HepG2, all cell lines with low Smad7 expression levels (HCC-M, HCC-T, PLC, Hep3B) exhibited a prolonged induction of pSmad2, while cell lines with high Smad7 levels, displayed more transient Smad2 phosphorylation. However, the latter do not show significant Smad7 induction by TGF-β, indicating that the TGF-β dependent negative feedback function of Smad7 is not controlling the duration of Smad2 phosphorylation. In contrast, induction of Smad7 correlates with CAGA reporter system activation by TGF-β which is low in HCC-M and HCC-T but high in PLC and Hep3B (see below) but not with Smad2 phosphorylation duration. We conclude, that basal Smad7 levels predetermine the cells sensitivity towards TGF-β induced cytostasis. Inducibility of Smad7 itself, however, is strictly dependent of Smad3 transcriptional activity and therefore high in cytostatic responsive cell lines. But this induced Smad7 is not abrogating or negatively controlling the cytostatic program of TGF-β.

Interestingly, Smad2 dependent ARE reporter activation was not correlated with Smad2 phosphorylation duration, but with cytostatic sensitivity, as reflected by strong activation of luciferase in PLC, HepG2 and HuH7 cells after TGF-β treatment (Figure 5A). Significant, but less activation is seen in Hep3B.

### Smad3/4 dependent CAGA-Luc reporter activation and target gene transcription correlate with cytostatic TGF-β effects

All cell lines display immediate induction of Smad3 phosphorylation upon 1h of TGF-β treatment. The induction is stable up to 48 hours in Hep3B, HCC-M, HLE, HuH7, HCC-T and PLC cells, while it is only transient in HepG2, HLF, HuH6, and FLC4 cells (Figure 5B, Figure S5). Surprisingly, Smad3 phosphorylation events were, in contrast to Smad2, not correlated to Smad7 expression. Also, no correlation to Smad7 induction by TGF-β (Figure 4A) was detectable. Because R-Smad phosphorylation only represents the first step in TGF-β signaling, it may not solely explain differences in the cytostatic TGF-β response among cell lines. Therefore, we additionally investigated induction of TGF-β/pSmad3 dependent transcription by measuring the relative CAGA reporter activity after 9h TGF-β treatment. HepG2, Hep3B, PLC and HuH7 cells exhibited relatively high CAGA-Luc-activity upon TGF-β treatment, while the other cell lines did not (Figure 5B). We found that the responsive cell lines have relatively little endogenous TGF-β and Smad7 (compare Figure 2A, Figure 5B). Most obviously, we demonstrated that CAGA reporter activation by TGF-β strongly correlated with sensitivity of the cell lines towards cytostatic TGF-β effects. Very intriguing, HCC-M and HCC-T display strong and prolonged TGF-β induced Smad3 activation, however very low if any CAGA reporter activation. In HCC-T cells, using immunofluorescence, we identified strong nuclear staining with an antibody that detects serine 208/213 phosphorylation at the linker region of Smad3 (Figure 5C). Such intrinsic linker phosphorylation was previously described to inhibit signaling of C-terminally phosphorylated Smad3 [18] and thus, may explain the controversy between Smad3 activation and transcriptional activity. Such mechanism however remains to be functionally proven.

As endogenous Smad7 levels are relatively low in TGF-β responsive cell lines and Smad7 *per se* is a TGF-β target gene of the early signature group, we compared the ability of TGF-β to induce the Smad7 promotor and Smad7 mRNA expression (Figure 4). We observed that TGF-β mediated Smad7 promoter activation perfectly reflected its mRNA induction, indicating transcriptional regulation of the expression. Furthermore, the level of Smad7 induction paralleled that of TGF-β induced CAGA reporter activation (PLC, HepG2, Hep3B, HuH7).

Other Smad3 dependent TGF-β target genes (Bim, PAI-1) were also investigated (Figure 4B). In line with its apoptotic function, Bim expression was upregulated upon TGF-β treatment in PLC, Hep3B and HuH 7 cell lines showing a cytostatic TGF-β response. PAI-1 expression also correlated to TGF-β sensitivity and was induced in PLC, Hep3B and HepG2.

### No coherent survival signaling status regulates cytostatic TGF-β effects

**Figure 6:**
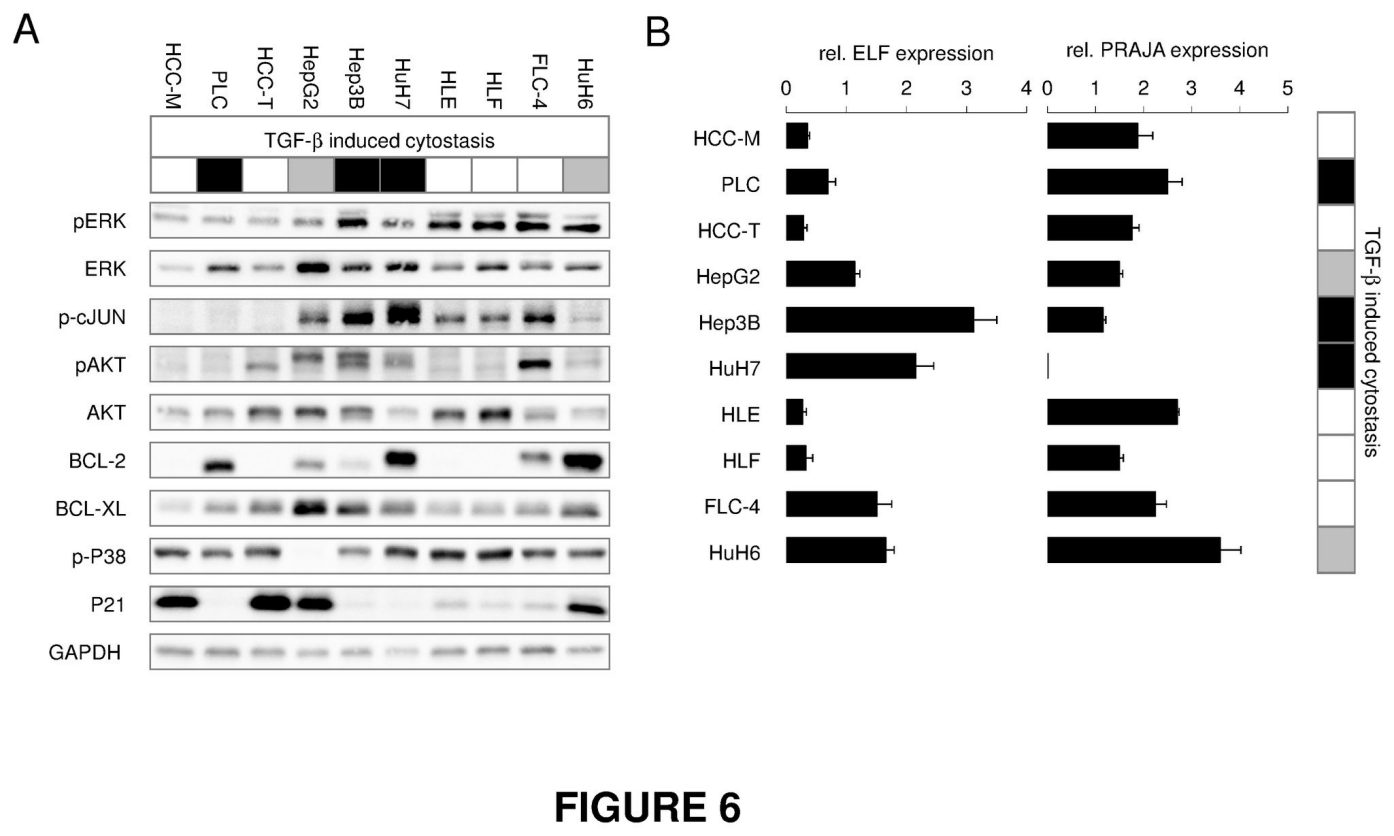
Endogenous expression of survival factors and Smad3 signaling modulators PRAJA and ELF in HCC cells. HCC cell lines were cultured in starvation medium for 24 h. (A) Western blot analysis was performed to evaluate expression levels of Akt, Bcl-2, Bcl-XL, p21 and GAPDH as loading control. Additionally, phosphorylation levels of ERK, c-Jun, Akt (lower band) and p38 were detected. (B) Basal PRAJA and ELF mRNA levels were quantified by Real time PCR analysis using rS18 as reference gene. Tables in (A) and (B) highlight cell lines responding to TGF-β with cell death and growth arrest in black. Cells which mainly show an inhibition of proliferation are marked as grey.

As balance between survival and cytostatic pathways plays an important role in determining the fate of hepatocytes and chronic liver disease progression, we asked whether the activity of classical survival pathways may explain differences in cytostatic responsiveness of HCC cell lines. Expression of BCL-2, BCL-XL, P21 and AKT as well as phosphorylation of P38, c-JUN, ERK and AKT do not exhibit any general link to TGF-β dependent cytostasis in the tested HCC cell lines (Figure 6A, Figure S6). However, induction of P21 expression (and to some extent downregulation of c-MYC) by TGF-β is a significant marker for TGF-β dependent cytostasis (see Figure 1A).

### Potential regulation of Smad3 signaling by ELF and PRAJA

It was reported that adaptor protein ELF is essential for Smad3 dependent TGF-β signaling and its down-regulation, e.g. by PRAJA mediated proteasomal degradation, can decrease Smad3 signaling and cause loss of the cytostatic response [8,19]. Thereby, loss of ELF could support gastric cancer and HCC development [20–22]. Testing this mechanism comparatively in HCC cell lines, we observed heterogeneous expression of both proteins. However, cytostatically responsive Hep3B and HuH7 cells expressed high levels of ELF and rather low levels of PRAJA (Figure 6B). In HepG2 and Huh6 showing a weaker cytostatic TGF-β response, ELF and PRAJA expression is intermediate. HCC-M, HCC-T, HLE and HLF do not show cytostasis upon TGF-β treatment and express relatively low ELF, but high PRAJA. Generally spoken, relative ELF/PRAJA ratios are higher in cytostatically responsive cell lines than in insensitive ones.

### RNAi with Smad3 conflicts TGF-β dependent cytostasis in TGF-β sensitive HCC cells

**Figure 7:**
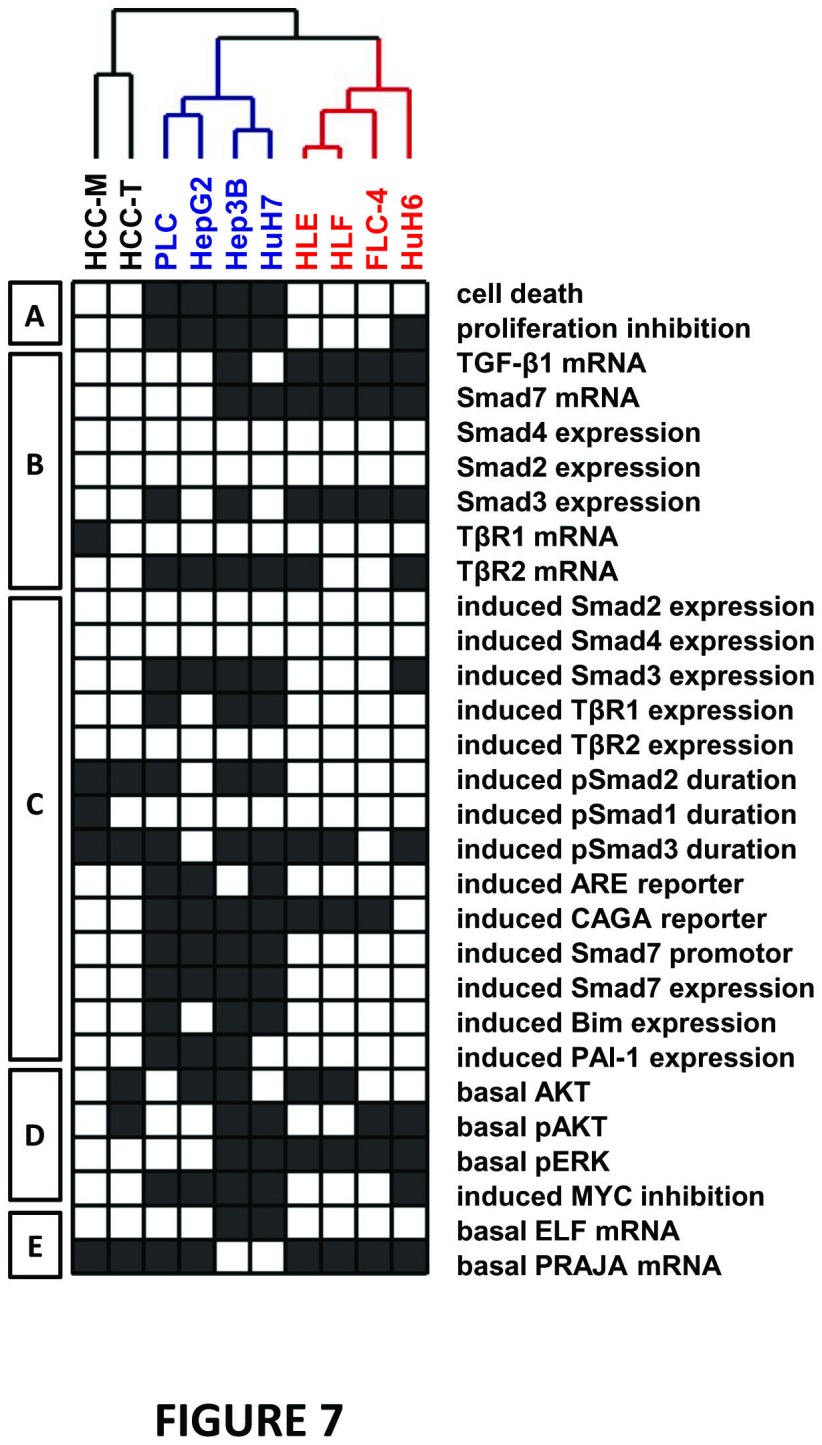
Hierarchical clustering analysis of HCC cell lines based on TGF-β/Smad signaling and cytostatic outcome. TGF-β related observations on the 10 HCC-derived cell lines (as summarized in Figure 9) were converted into an all-or-none fashion (black/white boxes) to produce a matrix which was organized by hierarchical clustering algorithm. Observations were divided into 5 broad categories (A-E, left side). (A) TGF-β dependent cytostasis (black boxes: cell death >5%; proliferation inhibition >50%), (B) basal TGF-β signaling (black boxes: 2^-ΔΔCt^ > 2.5 for all, but >4 for Smad3), (C) induced TGF-β signaling (black boxes rel. induction of expression 2^-ΔΔCt^ > 2, induction of CAGA and Smad7 promoter and expression > 2.8, induction of ARE reporter > 5 ; prolonged duration), (D) survival signaling (black boxes: >3-fold increased for pAKT total AKT, 6-fold increased for pERK; > 40% inhibition of c-MYC), (E) basal ELF and PRAJA expression (black boxes: 2^-ΔΔCt^ >2 and 2^-ΔΔCt^ >1.4 respectively). Based on the integrated above observations, clustering analysis unambiguously clustered the 10 cell lines. Beside HCC-M and HCC-T cell lines, 2 main groups were identified: group 1 included HepG2, PLC, Hep3B, HuH7 and group 2 included HLE, HLF, HuH6, FLC-4.

Hierarchical clustering summarized our biochemical results and confirmed three HCC cell line groups (Figure 7): (1) HepG2, Hep3B, HuH7, PLC; (2) HLE, HLF, FLC-4, HuH6 as well as (3) HCC-M plus HCC-T. The clusters differ in their sensitivity towards TGF-β induced cytostasis, which is correlated to different Smad7 and TGF-β expression levels, duration of induced Smad2 phosphorylation, Smad3 and Smad2 transcriptional activity, TβRII expression and inducibility of TβRI mRNA.

**Figure 8:**
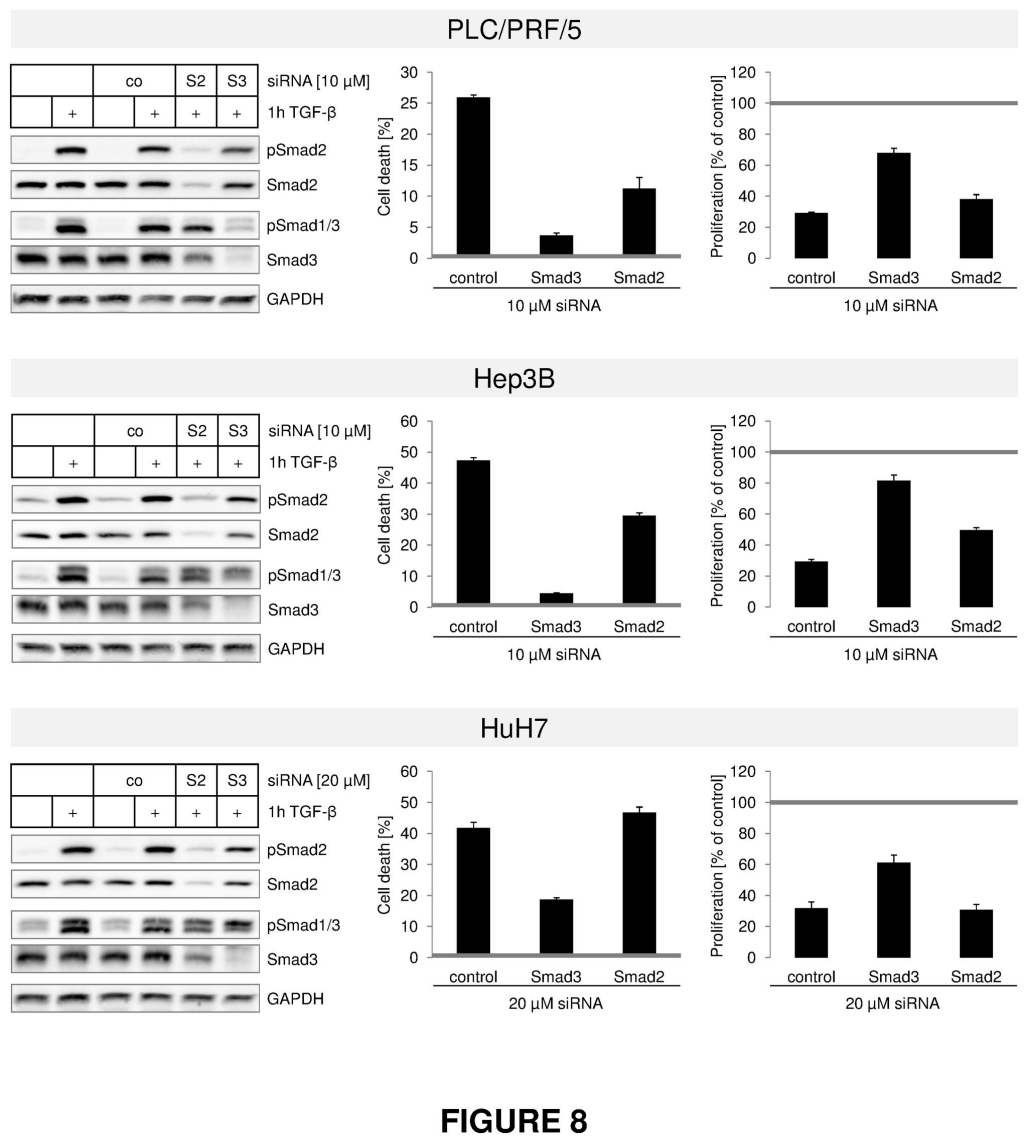
TGF-β induced cell death is Smad3 dependent. RNA interference technology was used to downregulate Smad2 (S2) and Smad3 (S3) in PLC/PRF/5 (upper panel), Hep3B (middle panel) and HuH7 (lower panel) cells. An unspecific siRNA sequence (co) was used as control. Knockdown was allowed to take place for 48 h. Afterwards, each setup was treated with or without 5 ng/ml TGF-β for 1 (Immunoblot) or 3 days (cell death and proliferation) (Left). Western blot analysis against phosphorylated and total Smad2 and Smad1/3 was performed to confirm successful knockdown. GAPDH was used as loading control (Middle). After 3 days with or without TGF-β, cell death rates were evaluated using an LDH assay. Untreated cells for each siRNA were defined as 0 (grey line). Filled bars show TGF-β treated samples (Right). LDH content of viable (adherent) cells was used to determine proliferation rates. TGF-β treated samples (filled bars) were related to untreated siRNA samples (grey line).

Taken together, the clusters demonstrate that disrupted Smad3 downstream signaling is required for loss of cytostatic TGF-β effects in liver cancer. Additionally, TGF-β strongly enhanced Smad3 expression and its transcriptional activity in cell lines with retained TGF-β mediated cytostasis. For functional proof of the critical role of Smad3 in TGF-β mediated cytostasis, we knocked down Smad3 or Smad2 in Hep3B, HuH7 and PLC, and investigated the resulting TGF-β effects on apoptosis and proliferation inhibition (Figure 8). In line with our hypothesis, we find that Smad3 knock down diminishes TGF-β induced cytostasis, while the effect of Smad2 knock down is comparably little. The fact that siRNA against Smad2 also reduces Smad3 expression to some extent may even direct the observed Smad2 knock down effects towards Smad3 function.

These results functionally confirm the predominant role of Smad3 in cytostatic outcome of TGF-β on liver parenchymal cells and indicate loss of Smad3 mediated downstream effects as critical for carcinogenic transdifferentiation.

## Discussion

TGF-β exhibits tumor suppressive functions in early liver disease (4). In later stages, including hepatocellular carcinoma, it may convert to tumor promotion by amputating cytostatic signaling branches and by facilitating EMT, migration and invasion. In our study, we comparatively investigated 10 HCC cell lines with regard to TGF-β signaling, its cytostatic effects and regulation.

**Figure 9:**
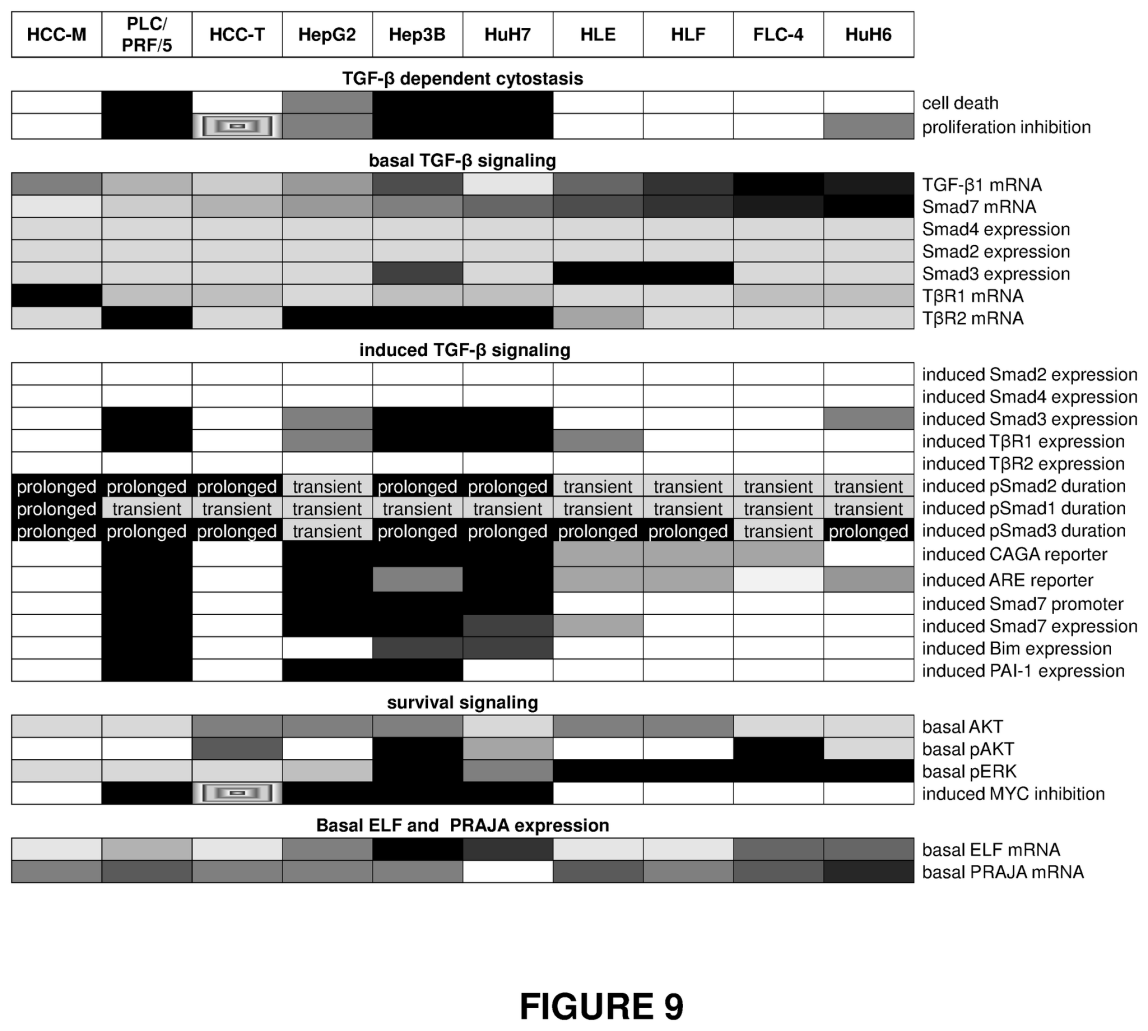
Overview of basal expression levels and TGF-β response in 10 different liver cancer cell lines. TGF-β dependent cytostasis, induced TGF-β signalling and inhibition of MYC expression: Here, the table gives an overview about TGF-β induced effects. The darker the field, the stronger is or, as for proliferation inhibition, the earlier occurs the described response. Basal TGF-β signalling, survival signalling and ELF and PRAJA expression: Basal expression and protein levels of various genes were analyzed. The results are interpreted with different gray scales with increasing darkness for higher expression levels. Scattered fields display cells which react contradictory to the described effect. The overview highlights some correlations found by the experiments described in Figures 1-6: a) Except for HuH6 cells, cell lines with a cytostatic response show similar responses: Strong TGF-β dependent increase in Smad3 and TβR1 expression and activation of Smad7 and Smad3 promotor activity. The latter one is mirrored in an induction of Smad3 target gene expression (Bim and PAI-1). b) Smad7 expression correlates to TGF-β1 mRNA, the duration of the Smad2 activation, but also to basal ERK phosphorylation. c) In many cases HCC-M and HCC-T cells show completely different features compared to cell lines with similar Smad7 expression.

As visualized in the comparative overview (Figure 9), our data suggest that HCC cell lines can be generally divided into 3 groups. This was confirmed with a hierarchical clustering approach integrating all observations related to TGF-β/Smad signaling and cytostatic outcome (Figure 7). The cluster discriminates the cell lines based on an unsupervised analysis. One group (PLC, HepG2, Hep3B, and HuH7) is responsive to TGF-β induced apoptosis and proliferation inhibition. These cells express relatively low endogenous levels of TGF-β and Smad7 and TGF-β treatment induced i) TβRI expression, ii) Smad3 expression without influencing phosphorylation duration, iii) Smad3 dependent transcription activation (CAGA reporter assay), iv) Smad7 promoter activity and Smad7 mRNA expression as well as v) by trend - long term Smad2 phosphorylation.

The second group (HLE, HLF, FLC-4, to some extent HuH6) behaves oppositely. Most importantly, they display i) loss of cytostatic effects upon TGF-β treatment (except late proliferation inhibition in HuH6 cells), ii) transient phosphorylation of Smad2 upon TGF-β treatment, iii) elevated endogenous ERK phosphorylation, iv) low induction of CAGA reporter and v) low Smad3 and TβRI and vi) high TGF-β1 and Smad7 expression. As HuH6 cells derive from hepatoblastoma instead of HCC cells, this may explain its outlying behaviour within this group in some aspects.

The third group comprising HCC-T and HCC-M that lack a cytostatic response despite strong intrinsic P21 expression, display some features of responsive cells like i) strong Smad3 phosphorylation, ii) low TGF-β1 and Smad7 expression, but controversially show iii) no CAGA- or ARE reporter activation, and iv) no TGF-β induced Smad7 promoter, Smad7, Bim or PAI-1 mRNA. We think that this finding is probably mainly due to the occurrence of R-Smad linker phosphorylation in these cells, as shown for HCC-T, which is able to hinder R-Smad transcriptional activity despite significant phosphorylation [12].

Many TGF-β signaling regulation mechanisms in healthy and damaged organs are described. Mutations in TGF-β signaling components are prominent in some cancer entities, including colon and pancreas [23], whereas this seems to be a rather rare event in HCC. Instead, major impact on downstream signaling regulation and switching the outcome of the pathway from tumor suppressive to tumorigenic seems to be central in HCC. Early studies describe upregulation of TGF-β in invasive HCC [24], low levels of TβRII in HCC with intrahepatic metastasis [25,26] and elevated levels of Smad7 in late stage HCC and other cancers [10,27,28]. We demonstrate that HCC cells insensitive for cytostatic TGF-β effects express high amounts of TGF-β and Smad7. Accordingly, we find Smad7 mRNA upregulation in 68.5% of 143 investigated human HCC tumors as compared to surrounding non-tumorous tissue (manuscript in preparation). Thus, high intrinsic Smad7 mRNA levels reflect one mechanism how HCC cells evade Smad3 dependent cytostatic TGF-β effects to facilitate disease progression. This is also reflected by previous investigations, where ectopic Smad7 expression blunted TGF-β induced apoptosis in Hep3B cells and Huh7 cells [29,30].

In contrast to Smad3, duration of Smad2 phosphorylation correlated to TGF-β sensitivity in cell lines, indicating distinct regulation and function of Smad2 and Smad3 in liver cells. An *in vivo* study on the different roles of Smad2 and 3 [31] demonstrates that hepatocytes deficient in Smad2 spontaneously acquire features characteristic of epithelial-to-mesenchymal transition (EMT), and further that Smad2 is not required for TGF-β stimulated growth inhibition in hepatocytes. The authors speculate that Smad2 is anti-metastatic during carcinogenesis, which is in line with loss of Smad2 phosphorylation in late rat HCC [32]. Accordingly, we find very transient Smad2 phosphorylation in cytostatically insensitive cell lines (HLE, HLF, FLC-4, HuH6) of which at least 2 (HLE and HLF) are invasive ( [33,34] and unpublished observations (HLE, HLF and FLC-4)).

ELF7/ β-Spectrin and PRAJA offer another TGF-β regulation system relevant in HCC [8,35]. ELF is a cytoplasmic cofactor required for correct subcellular localization of Smad3 and Smad4, while PRAJA marks ELF for proteasomal degradation, thus negatively interfering with TGF-β signaling. Except for PLC and FLC-4, our data support such hypothesis as further potential mechanism in HCC. In Hep3B and HuH7 cells, both sensitive to Smad3 dependent cytostasis, ELF is highly expressed, while PRAJA is present in low amounts. In Huh6 and HepG2, medium amounts of ELF and PRAJA correlate with low but still significant cytostatic TGF-β response. HCC-M, HCC-T, HLE and HLF displaying low ELF and high PRAJA expression are lacking the TGF-β cytostatic response. Since ELF acts downstream of R-Smad phosphorylation, its loss does not interfere with R-Smad activation but uncouples the latter from transcriptional regulation. Since several cell lines display strong Smad3 phosphorylation without significant CAGA-luc or Smad7 expression induction, our data further support such mechanism as relevant in HCC (Figures 4, 5B, 6B). However, in PLC and FLC-4, one responsive and one insensitive cell line, relative ELF and PRAJA expression levels do not explain cytostatic behaviour on their own, arguing for another mechanism to be responsible for regulation. However, in any case, functional and more importantly causal links still need to be demonstrated.

Hepatocyte plasticity and EMT are important constituents for liver disease dissolvement or progression [36–41]. When shutting down cytostatic TGF-β effects, survival pathways like pERK and pAKT dependent cascades dominate the delicate balance of cytostasis or survival in liver cells. As CAGA reporter gene activation but not Smad3 phosphorylation is affected in correlation to TGF-β induced cytostasis, our data indicate an intracellular regulation of cytostatic responsiveness downstream of receptor activation and Smad3 phosphorylation. It might be reasonable to argue, that in HLE, HLF, FLC-4 and HuH6, a shift from canonical Smad to noncanonical Smad signaling (e.g. Akt, ERK, JNK pathways) occurred upon TGF-β treatment probably due to high endogenous Smad7 levels. Accordingly, we show that HCC cell lines, which do not react cytostatically upon TGF-β display high amounts of pERK and, except for HuH6 cells, p-cJUN. However, also some cell lines, which are sensitive towards TGF-β dependent cytostasis show relatively high pERK and p-cJUN levels (Hep3B, HuH7) again implying a complex regulation network to distinguish between cytostatic and survival effects in HCC cell lines. Thus, it was shown in Hep3B, PLC/PRF/5 and Huh7 that TGF-β may induce apoptosis or survival, depending on absence or presence of EGFR ligands [42,43]. However, HepG2 cells with a mutated Ras/ERKs pathway exhibit apoptosis resistance (reduced rate) that cannot be rescued through EGFR blockade.

HCC-M and HCC-T display a distinct behaviour, and therefore, are representative for a third and very interesting group of HCC cell lines with respect to TGF-β (Figure 7). HCC-M and HCC-T, both display long term phosphorylation of all R-Smads tested (Smad1, 2, 3) upon TGF-β treatment but no reporter gene activation and cytostatic response. Rather low Smad7 levels suggest further mechanisms of signaling regulation. One possibility low ELF but high PRAJA expression, which deregulates Smad3 localization and activity. As no activation of the CAGA reporter assay was achieved by TGF-β treatment, we also speculate that IGFBP2 via activation of Akt and/or Yap mediated stabilization of Smad7, as recently described for cancer stem cells (Machida et al. unpublished), might interfere with cytostatic TGF-β/Smad signaling. Another possibly applicable mechanism was demonstrated by Matsuzaki and co-workers, showing that in patients with chronic liver disease progression, JNK-dependent linker phosphorylation of Smad3 in hepatocytes occurs, which subsequently interferes with cytostatic R-Smad downstream signaling [12,44,45]. Indeed, HCC-M and HCC-T show high levels of linker phosphorylation of Smad3 and nuclear staining, making the relevance of such mechanism probable in these HCC cell lines and as well in human disease, since preliminary data with HCC patient samples suggest the occurrence of Smad3L-phosphorylation in late stage disease (data not shown), which now will be systematically investigated.

While liver research successfully makes use of cell lines since a long time, many contrary results on cellular processes have been reported over time. In this regard, the presented data will impact the understanding of human hepatocarcinogenesis by providing a robust rationale for the use of relevant HCC cell lines to model specific aspects of HCC onset and progression. For the first time, we provide comparative, correlative and relative information comprising mechanistic details about TGF-β action and regulation in an exhaustive set of human HCC cell lines. These new data extend the first array based characterization of early and late TGF-β signatures in HCC [17]. Our data strongly suggest that the shift between tumor suppressive and tumor promoting TGF-β effects involves different regulation of Smad3 dependent transcription, TβRI expression, Smad2 signaling duration, and endogenous TGF-β/Smad7/TβRII levels. Further, our results exemplify the diversity of mechanisms involved in the regulation of TGF-β effects, even when investigating one specific tumor entity, in this case HCC. While the exact regulation of cytostatic TGF-β sensitivity appears to be complex, diverse and context specific, the cell lines could be assorted into three groups. In early stages of chronic liver disease and onset of HCC development, TGF-β is described to fulfil tumor suppressive and cytostatic functions, well represented by induction of apoptosis and proliferation inhibition by TGF-β in PLC, HepG2, Hep3B and HuH7. HLE, HLF, FLC-4 (and Huh6) cells on the other hand more robustly represent late stage disease with lost cytostatic TGFβ signaling. A third group comprises HCC-T and HCC-M, also representing late stage disease, but displaying counter regulation of TGF-β signaling via linker phosphorylation, which is reflected in a completely unusual setting in regard to our biochemical analyses.

As targeting TGF-β signaling is still under discussion for cancer treatment, our data will also influence HCC drug development. Future research needs to determine the exact time point of the switch from cytostatic to tumor promoting TGF-β effects finally allowing selection of patients relevant for anti-TGF-β therapies. Further studies on EMT and migrative, invasive features of the cell lines are currently ongoing. Animal studies will then determine beneficial and harmful time points to interfere with TGF-β signaling during HCC development making validation in patients the most important next step.

## Supporting Information

Table S1 **Primary and secondary antibodies used for immunoblot analysis.** Antibodies were obtained from Cell Signaling (Danvers, MA, USA), Santa Cruz Biotechnology (Santa Cruz, California, USA.), Sigma Aldrich (St. Louis, Missouri, USA), BD Bioscience (Heidelberg, Germany) or Epitomics (Burlingame, California, USA). After blocking the membrane with 5% non fat milk powder in TBST, the membrane was incubated with the first antibody over night at 4 °C applying careful agitation. After removal of excessive antibodies, the membrane was incubated with the second antibody in TBST for 3-4 h at room temperature.
(TIF)

Table S2 **Origin of the cell line, the original patient characteristics (age, sex, tumour stage), cell lines passages.**
(TIF)

Figure S1 **18S rRNA is a suitable reference gene for real time PCR analysis of the used liver cancer cell lines.** (A) 18S rRNA is equally expressed between the different liver cancer cell lines. Real Time PCR experiments were performed using 18S rRNA as reference gene. An analysis of the Ct values of 18S rRNA revealed minor fluctuations confirming the suitability of 18S rRNA as reference gene. Results are shown as mean +/- SE for 3 (HCC-M and HuH6) to 4 independent experiments. (B) TGF-beta treatment of HCC cell lines resulted in no or negligible changes of expression of 18S rRNA in liver cancer cell lines. The diagram shows the mean deviation (ΔCt) of 18S rRNA Ct values from control and TGF-beta treated samples (24 h) of the same cell line. Results are presented as the mean +/- SE of 2-3 independent experiments.
(TIF)

Figure S2 **Densitometric analysis (ImageJ software) of Western Blots in Figure 1 and corresponding repetitive experiments.** Results are shown as mean +/- SE of the indicated numbers of independent experiments.
(TIF)

Figure S3 **Densitometric analysis (ImageJ software) of Western Blots presented in Figure 2 and corresponding repetitive experiments.** Results are shown as mean +/- SE of the indicated numbers of independent experiments.
(TIF)

Figure S4 **Densitometric analysis (ImageJ software) of Western Blots presented in Figure 3 and corresponding repetitive experiments.** Results are shown as mean +/- SE of the indicated numbers of independent experiments.
(TIF)

Figure S5 **Densitometric Analysis (ImageJ software) of Western Blots in Figure 5 and corresponding repetitive experiments.** Results are shown as mean +/- SE of the indicated numbers of independent experiments.
(TIF)

Figure S6 **Densitometric analysis (ImageJ software) of Western Blots in Figure 6 and corresponding repetitive experiments.** Results are shown as mean +/- SE of the indicated numbers of experiments.
(TIF)
