# Correction: Comparative Analysis of TGF-β/Smad Signaling Dependent Cytostasis in Human Hepatocellular Carcinoma Cell Lines

**DOI:** 10.1371/journal.pone.0095952

**Published:** 2014-05-08

**Authors:** The PLOS ONE Staff

The authors have provided the following notice of correction regarding the contamination of two cell lines used in their study. Corrected versions of all figures and supporting information files can be found below or attached to this notice.

Cell line authentication (STR analysis, Leibniz-Institute DSMZ GmbH) identified cell lines named HCC-M and HCC-T in the publication ’Comparative analysis of TGF-β/Smad signaling dependent cytostasis in human hepatocellular carcinoma cell lineś (PLOS one, 2013, 8 (8), e72252) as **HeLa** cell populations. Experiments putatively using HCC-M and HCC-T cells were described in the main body and the figures of the manuscript several times.

Generally spoken, these results cannot be assigned to HCC-M or HCC-T specific characteristics, but need to be considered to serve as experimental controls. However, and most importantly, our finding directly strengthens the conclusion of the publication that HCC cell lines can be assorted into two clusters according to their sensitivity for TGF-β dependent cytostatic effects. Additionally, we can now state, that there is no outlying cell line in our study combining characteristics of both groups. In fact our results rather suggest that in HCC cell lines, TGF-β signaling can be classified exactly using the criteria applied in the present paper. In contrast, HeLa cells which originate from a different tumor entity (cervical cancer) display a completely different TGF-β signaling pattern than HCC cell lines.

To further validate robustness of our findings with the other cell lines used in the study, we did confirm identity of those. Huh7, HLE, HLF, HepG2 and PLC/PRF/5 were purchased from major cell banks by SFB/TRR77 and provided to us right before the study was performed. STR analysis of Hep3B, HepG2, HLE, Huh6 and FLC-4 confirmed the correct identity. Yet, we need to note that FLC-4 was identified as JHH-4, as a reference STR profile for FLC-4 is not available since the cell line is not deposited at one of the major cell banks. However, JHH-4 is the parental cell line of FLC-4, which was obtained by starvation mutation from JHH-4 (HUMAN CELL, Vol. 1, No. 1, p.98-100, 1988), and thus explains the analogous STR analysis results. Finally, we could exclude the possibility that the HeLa cross contamination of HCC-M and HCC-T occurred in our laboratory. Assessment of a new source of cells directly obtained from the originally providing laboratory (Prof. Dr. Hidetsugu Saito, Japan) by STR analysis, showed the same HeLa cross contamination. Of course, we informed the laboratory of origin about this finding.

**Figure 1:**
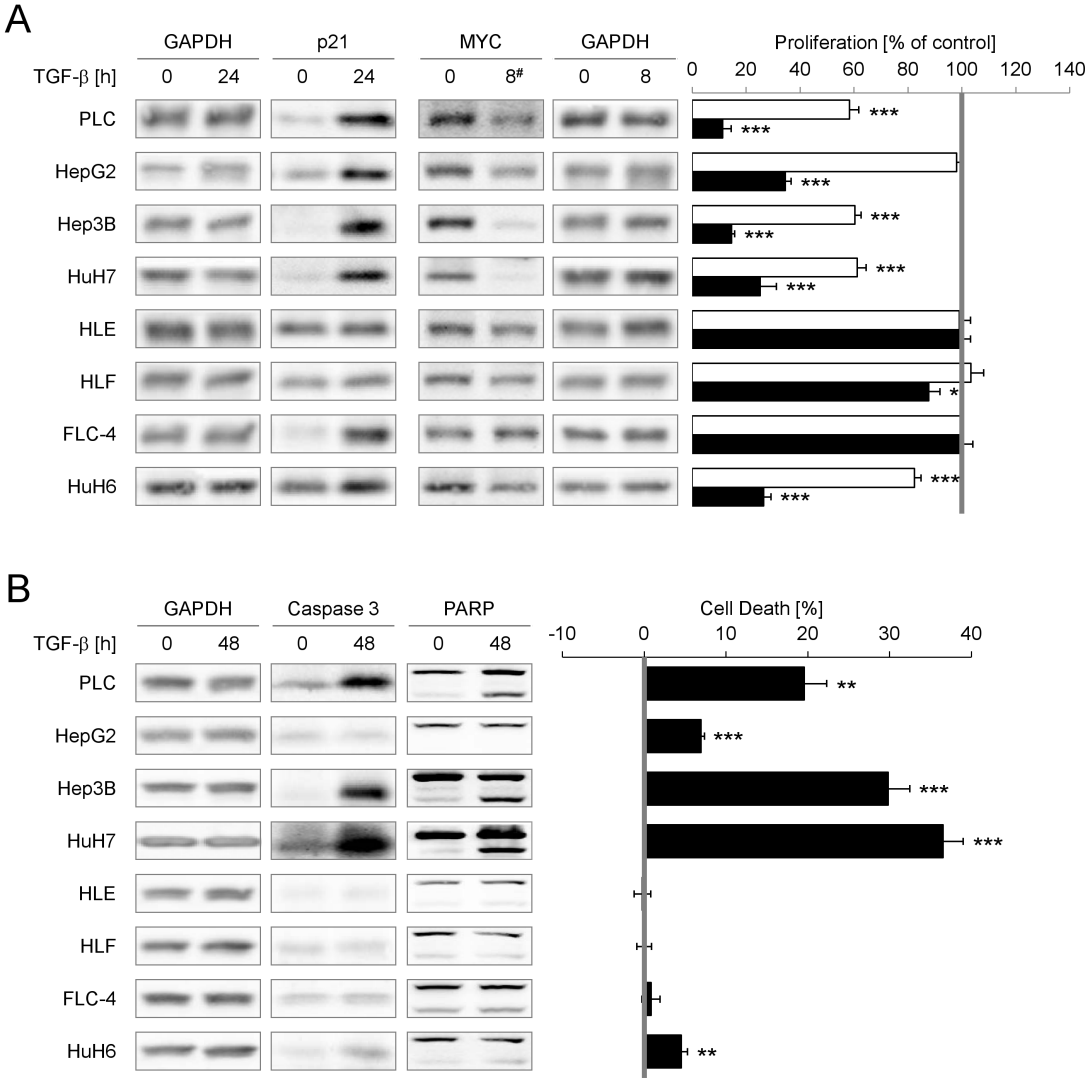
TGF-β induces cell death and/or inhibits proliferation in PLC, HepG2, HuH6, Hep3B and HuH7. (A) Left side: HCC cell lines were treated with 5 ng/ml TGF-β for indicated time points. Changes in c-MYC and P21 expression were detected using Western blot analysis with GAPDH as loading control. As HCC-T cells show a delayed TGF-β response, c-MMYC was induced after 72 h of TGF-β treatment. Right side: After TGF-β treatment for 0 (grey line), 2 (white bars) or 6 days (black bars) cell proliferation was evaluated by MTT assays. Treated samples were normalized to the corresponding control. (B) Left side: Western blot analysis of PARP and Caspase 3 cleavage using GAPDH expression as loading control after 0 and 48 h TGF-β treatment. In the case of HuH7 cells, the control sample was treated with TGF-β for 3 h. Right side: Cell death induced by 5 ng/ml TGF-β over 72 h (filled bars) was quantified by detecting LDH release normalized to total amount of LDH. Untreated samples were defined as 0 (grey line). Significant differences are indicated as * p < 0.05, ** p < 0.01 and *** p < 0.001 (Student’s t test).

**Figure 2:**
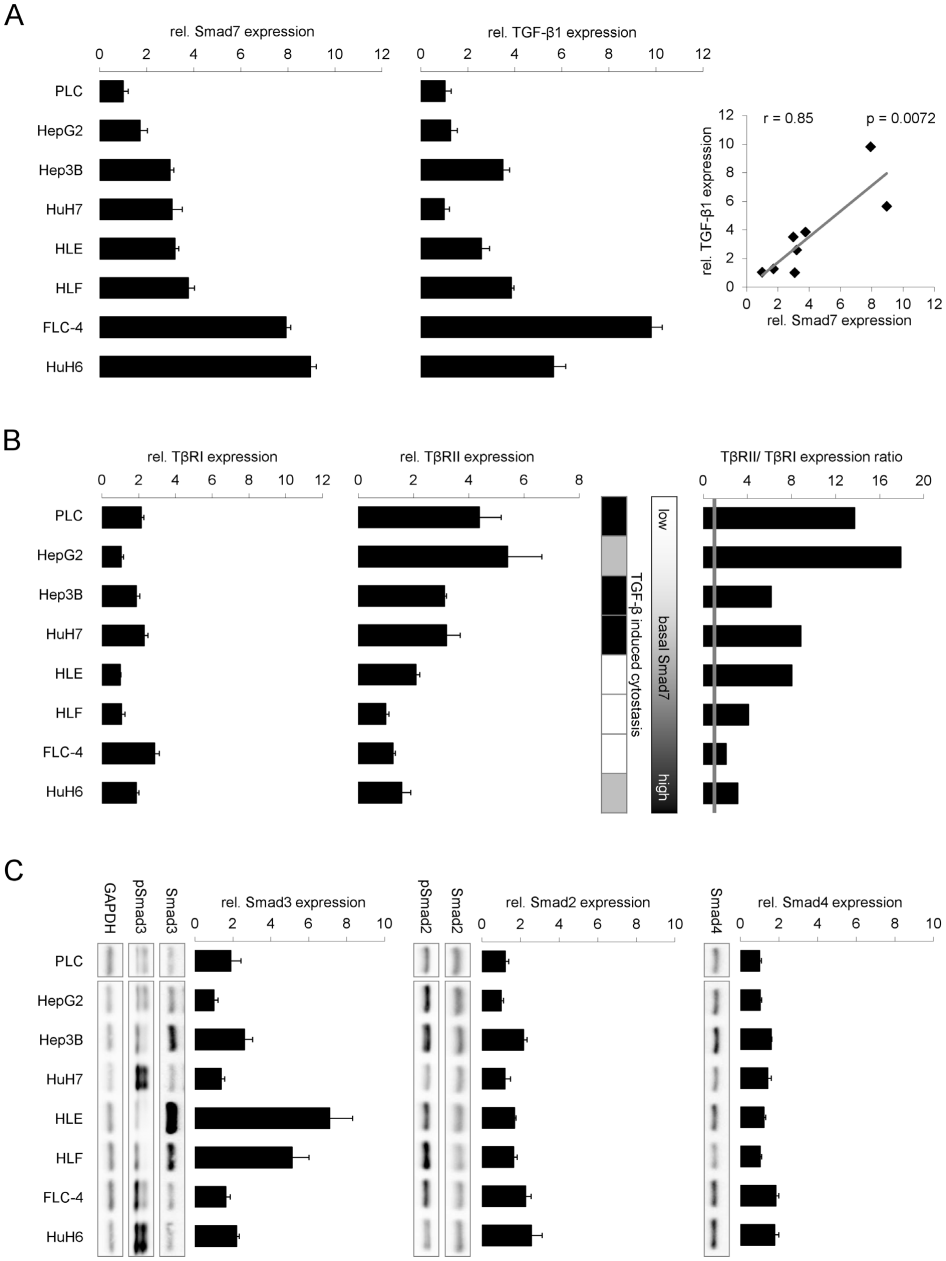
Expression levels of TGF-β/Smad signaling components in HCC cell lines. Relative TGF-β1 and Smad7 (A), TGF-β receptor I (TβRI) and II (TβRII) (B) and Smad2, Smad3, and Smad4 (C) expression levels were detected using real time PCR. Expression of 18s rRNA was used as reference and results were analyzed with the ΔΔCt method. A strong correlation of Smad7 and TGF-β1 expression was identified by calculating the Pearson coefficient (r  =  0.87; p  =  0.0011) (A). Further, TβRII expression (filled bars) was generally increased compared to the expression of TβRI (grey line) in the same cell line (B, right). Additionally, total and phosphorylated Smad2 and Smad3 as well as basal Smad4 protein levels were evaluated by Western Blot using GAPDH as loading control (C).

**Figure 3:**
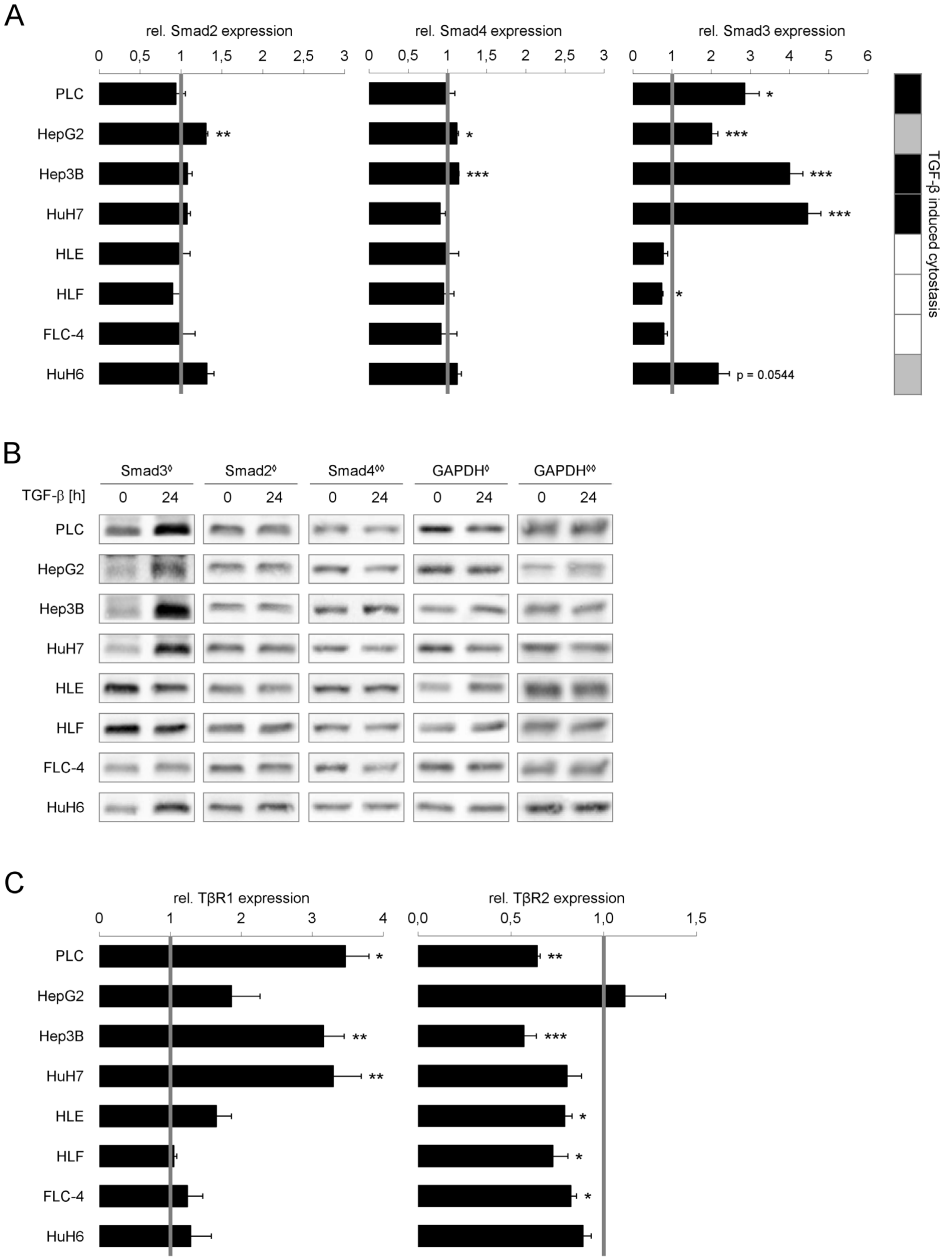
Induction of Smad3 and TβRI expression correlates with cytostatic responsiveness upon TGF-β treatment. Cells were cultured with or without 5 ng/ml TGF-β for 24 h. Changes in Smad2, Smad3 and Smad4 levels after TGF-β treatment were evaluated by (A) Real Time PCR and (B) Western Blot analysis using 18S rRNA or GAPDH as reference genes. **^◊^** and **^◊ ◊^** indicate which GAPDH belongs to Smad2 and Smad3 or Smad4, respectively. (C) TGF-β dependent expression levels of TGF-β Receptor I (TβRI) and II (TβRII) were detected and correlated against untreated samples using real time PCR with 18S rRNA as reference gene. Untreated samples are shown as grey lines, while filled bars display TGF-β treated samples. Significant differences are indicated as * p < 0.05, ** p < 0.01 and *** p < 0.001 (Student’s t test).

**Figure 4:**
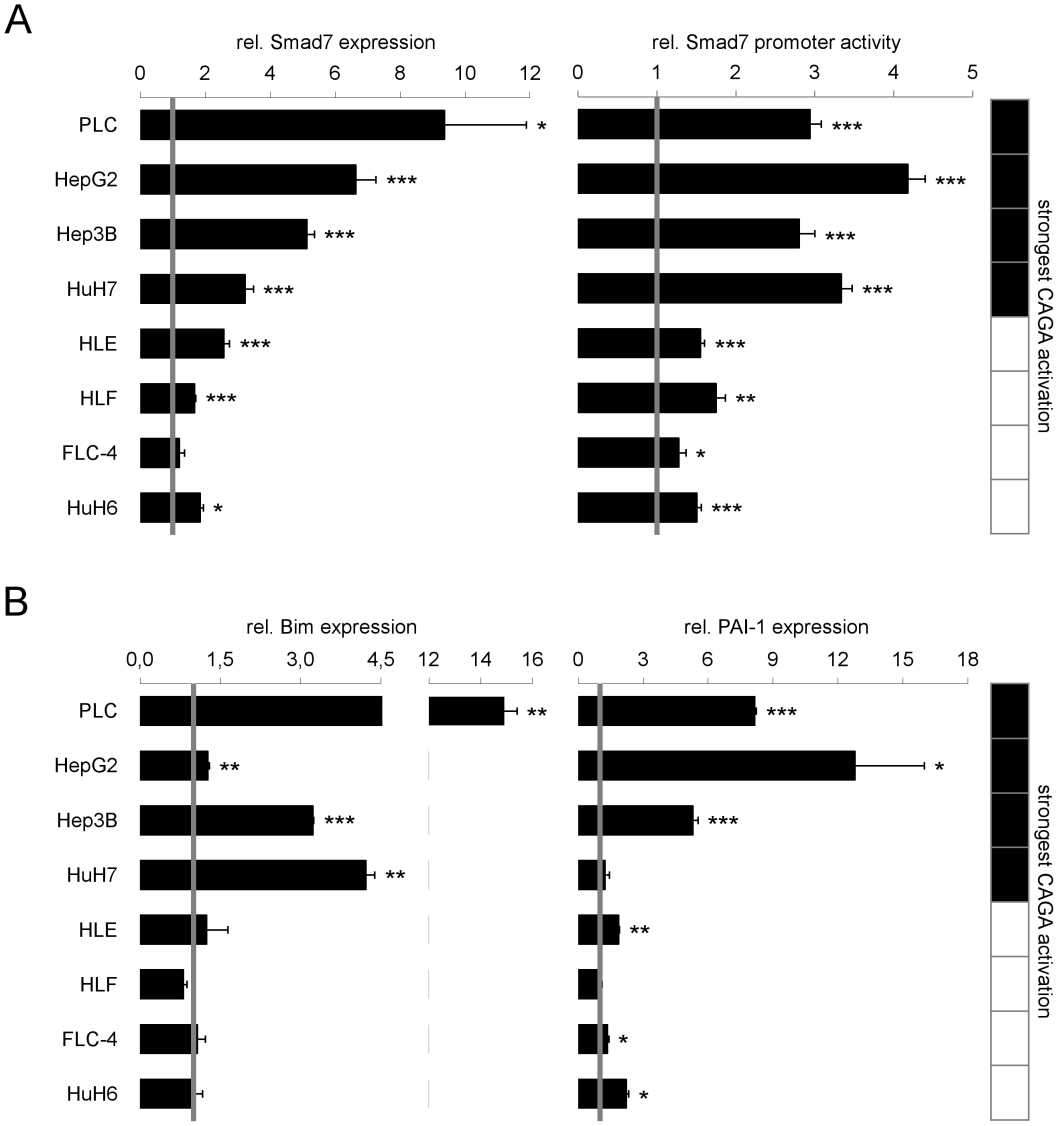
Induction of Smad7 expression by TGF-β correlates with cytostatic responsiveness. (A) Left side: Cells were treated with or without TGF-β for 2 h. Smad7 expression and 18S rRNA levels as reference gene were detected by real time PCR. Right side: For evaluation of transcriptional activity, HCC cell lines were transfected with a construct containing a luciferase gene under control of the Smad7-promotor and treated with TGF-β for 6 h. Treated samples were correlated to untreated controls. (B) Changes in expression levels of Smad3 target genes Bim and PAI-1 were detected after 2 h (PAI-1) or 24 h (Bim) using real time PCR analysis with rS18 as reference gene. The tables highlight cell lines with highest CAGA activity (black fields). Significant differences are indicated as * p < 0.05, ** p < 0.01 and *** p < 0.001 (Student’s t test).

**Figure 5:**
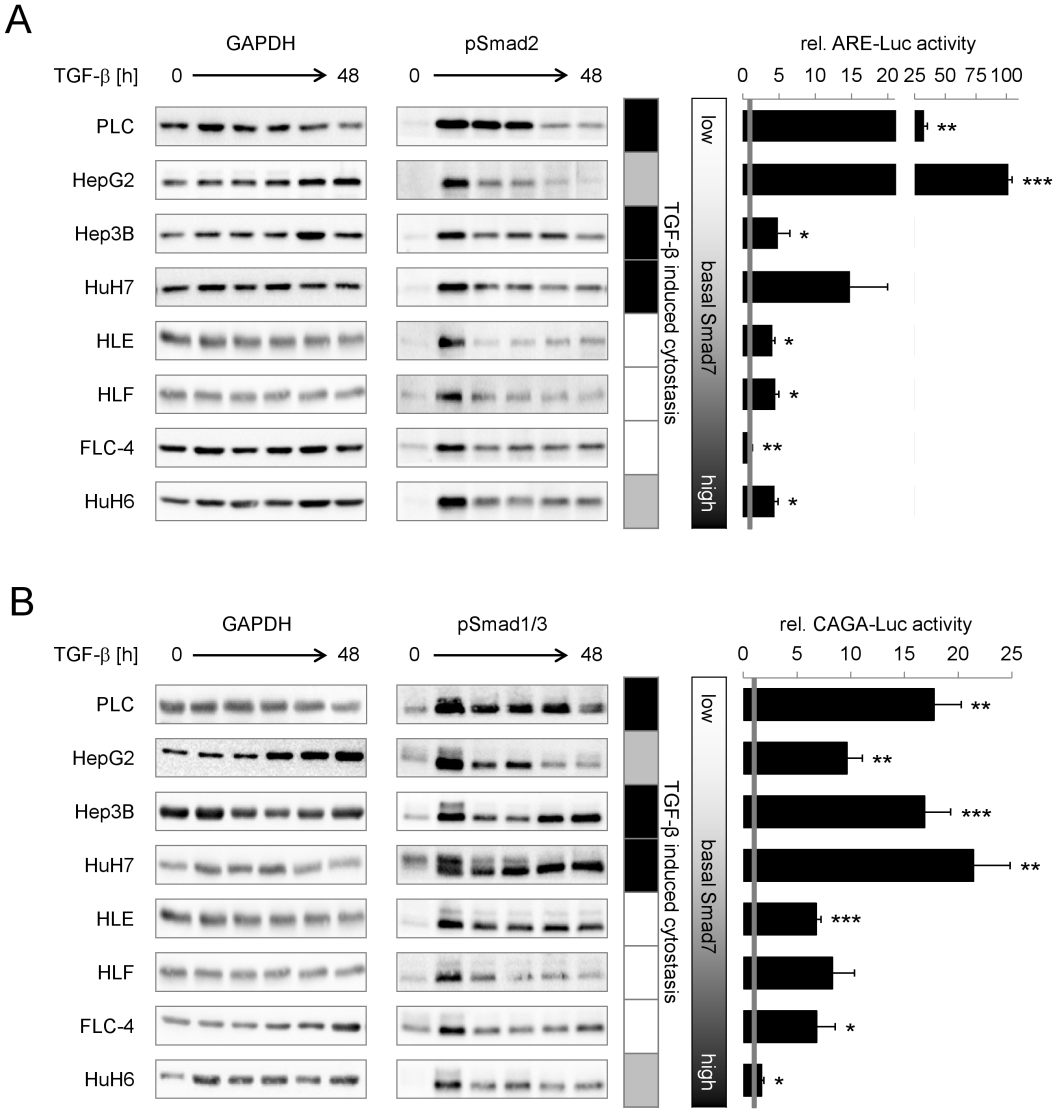
While Smad2 phosphorylation duration correlates to TGF-β and Smad7 expression, cytostatic responsiveness relates to CAGA-reporter-activation. HCC cell lines were treated with 5 ng/ml TGF-β for 0, 1, (3), 7, 24 and 48 h. (A) Left side: Western blot analysis of Smad2 phosphorylation and GAPDH expression. Right side: To evaluate TGF-β dependent transcriptional activity of Smad2, cells were transfected with plasmids carrying a luciferase gene under control of the activin response element (ARE) and additionally with a FAST-1 expression construct. Luciferase activity of cells treated with 5 ng/ml TGF-β for 9 h was correlated to untreated control samples. (B) Left side: Western blot analysis of Smad1/3 phosphorylation (1: upper/3: lower band); loading control GAPDH. Right side: TGF-β dependent transcriptional activity of Smad3 was evaluated 9 h after TGF-β treatment in cells infected with an adenovirus carrying a luciferase gene controlled by a CAGA response element. Treated samples were correlated to the corresponding untreated control. In the table, cell lines marked as black, show TGF-β induced cell death and growth inhibition whereas cells marked as grey mainly react with the latter one. The gradient from white to black displays increasing basal Smad7 expression levels. (C) Immunofluorescent detection of linker phosphorylated Smad3 in uninduced HCC-T cells. Red fluorescence indicates nuclear localization of pSmad3L. Blue staining indicates nuclei stained with DRAQ5. Significant differences are indicated as * p < 0.05, ** p < 0.01 and *** p < 0.001 (Student’s t test).

**Figure 6:**
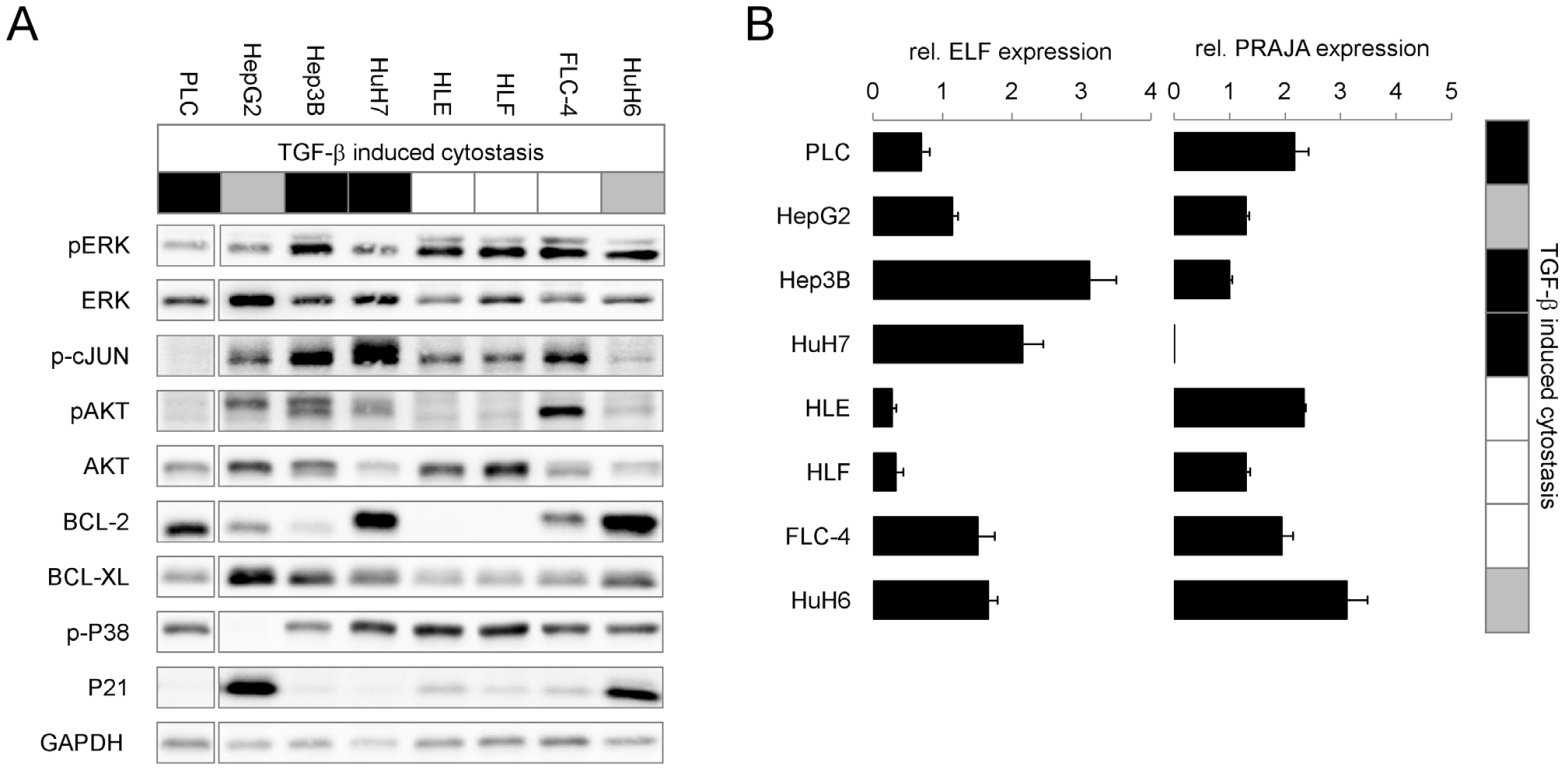
Endogenous expression of survival factors and Smad3 signaling modulators PRAJA and ELF in HCC cells. HCC cell lines were cultured in starvation medium for 24 h. (A) Western blot analysis was performed to evaluate expression levels of Akt, Bcl-2, Bcl-XL, p21 and GAPDH as loading control. Additionally, phosphorylation levels of ERK, c-Jun, Akt (lower band) and p38 were detected. (B) Basal PRAJA and ELF mRNA levels were quantified by Real time PCR analysis using rS18 as reference gene. Tables in (A) and (B) highlight cell lines responding to TGF-β with cell death and growth arrest in black. Cells which mainly show an inhibition of proliferation are marked as grey.

**Figure 7:**
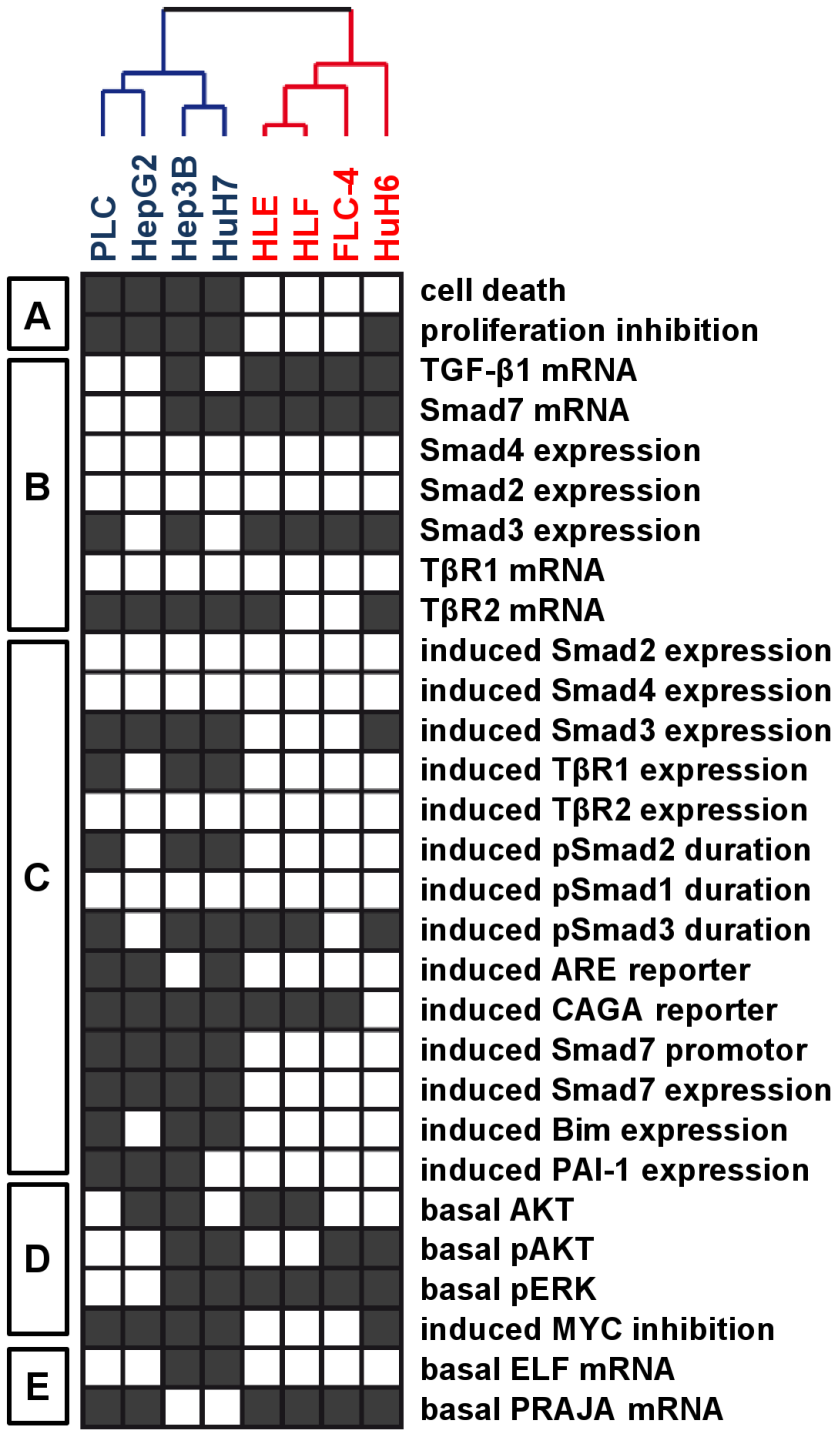
Hierarchical clustering analysis of HCC cell lines based on TGF-β/Smad signaling and cytostatic outcome. TGF-β related observations on the 10 HCC-derived cell lines (as summarized in Fig.9) were converted into an all-or-none fashion (black/white boxes) to produce a matrix which was organized by hierarchical clustering algorithm. Observations were divided into 5 broad categories (A-E, left side). (A) TGF-β dependent cytostasis (black boxes: cell death >5%; proliferation inhibition >50%), (B) basal TGF-β signaling (black boxes: 2^-ΔΔCt^>2.5 for all, but >4 for Smad3), (C) induced TGF-β signaling (black boxes rel. induction of expression 2^-ΔΔCt^> 2, induction of CAGA and Smad7 promoter and expression > 2.8, induction of ARE reporter > 5 ; prolonged duration), (D) survival signaling (black boxes: >3-fold increased for pAKT total AKT, 6-fold increased for pERK; > 40 % inhibition of c-MYC), (E) basal ELF and PRAJA expression (black boxes: 2^-ΔΔCt^ >2 and 2^-ΔΔCt^ >1.4 respectively). Based on the integrated above observations, clustering analysis unambiguously clustered the 10 cell lines. Beside HCC-M and HCC-T cell lines, 2 main groups were identified: group 1 included HepG2, PLC, Hep3B, HuH7 and group 2 included HLE, HLF, HuH6, FLC-4.

**Figure 8:**
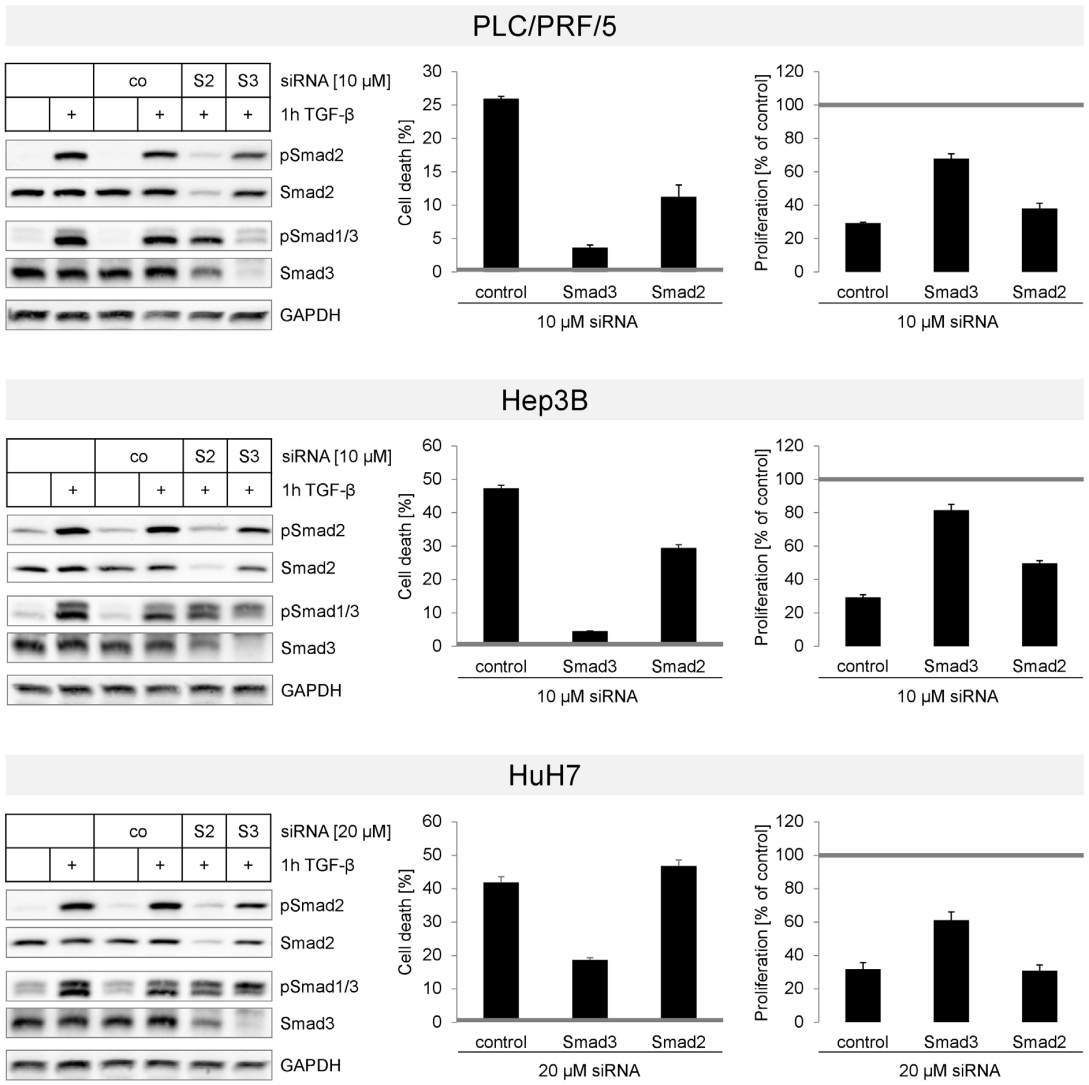
TGF-β induced cell death is Smad3 dependent. RNA interference technology was used to downregulate Smad2 (S2) and Smad3 (S3) in PLC/PRF/5 (upper panel), Hep3B (middle panel) and HuH7 (lower panel) cells. An unspecific siRNA sequence (co) was used as control. Knockdown was allowed to take place for 48 h. Afterwards, each setup was treated with or without 5 ng/ml TGF-β for 1 (Immunoblot) or 3 days (cell death and proliferation). (Left) Western blot analysis against phosphorylated and total Smad2 and Smad1/3 was performed to confirm successful knockdown. GAPDH was used as loading control. (Middle) After 3 days with or without TGF-β, cell death rates were evaluated using an LDH assay. Untreated cells for each siRNA were defined as 0 (grey line). Filled bars show TGF-β treated samples. (Right) LDH content of viable (adherent) cells was used to determine proliferation rates. TGF-β treated samples (filled bars) were related to untreated siRNA samples (grey line).

**Figure 9:**
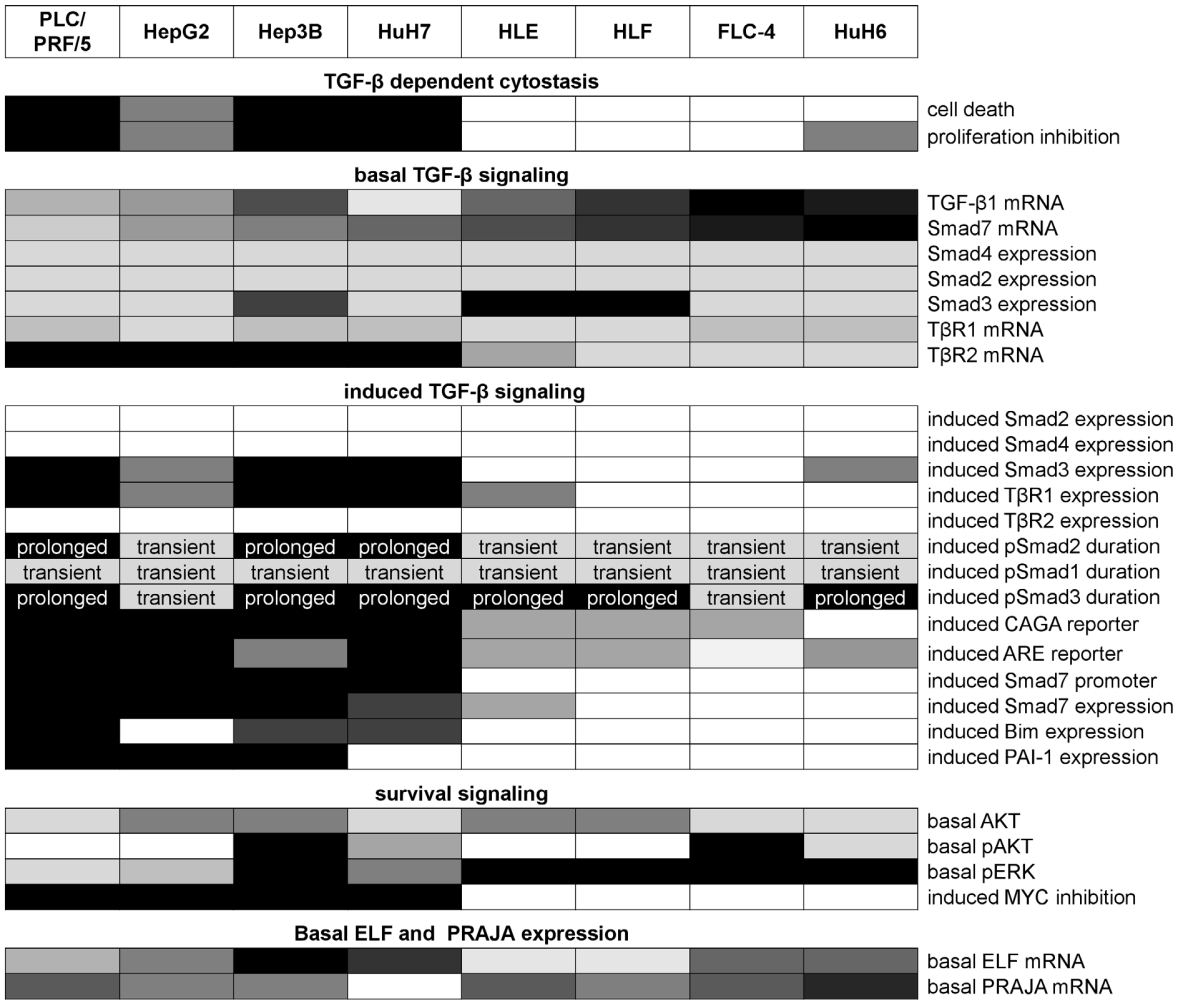
Overview of basal expression levels and TGF-β response in 10 different liver cancer cell lines. TGF-β dependent cytostasis, induced TGF-β signalling and inhibiton of MYC expression: Here, the table gives an overview about TGF-β induced effects. The darker the field, the stronger is or, as for proliferation inhibition, the earlier occurs the described response. Basal TGF­-β signalling, survival signalling and ELF and PRAJA expression: Basal expression and protein levels of various genes were analyzed. The results are interpreted with different gray scales with increasing darkness for higher expression levels. Scattered fields display cells which react contradictory to the described effect. The overview highlights some correlations found by the experiments described in Figure 1-6: a) Except for HuH6 cells, cell lines with a cytostatic response show similar responses: Strong TGF-β dependent increase in Smad3 and TβR1 expression and activation of Smad7 and Smad3 promotor activity. The latter one is mirrored in an induction of Smad3 target gene expression (Bim and PAI-1). b) Smad7 expression correlates to TGF-β1 mRNA, the duration of the Smad2 activation, but also to basal ERK phosphorylation. c) In many cases HCC-M and HCC-T cells show completely different features compared to cell lines with similar Smad7 expression.

## Supporting Information

Table S1 **Primary and secondary antibodies used for immunoblot analysis.** Antibodies were obtained from Cell Signaling (Danvers, MA, USA), Santa Cruz Biotechnology (Santa Cruz, California, USA.), Sigma Aldrich (St. Louis, Missouri, USA), BD Bioscience (Heidelberg, Germany) or Epitomics (Burlingame, California, USA). After blocking the membrane with 5 % non fat milk powder in TBST, the membrane was incubated with the first antibody over night at 4 °C applying careful agitation. After removal of excessive antibodies, the membrane was incubated with the second antibody in TBST for 3-4 h at room temperature.
(TIF)

Table S2 **Origin of the cell line, the original patient characteristics (age, sex, tumour stage), cell lines passages.**
(DOCX)

Figure S1 **18S rRNA is a suitable reference gene for real time PCR analysis of the used liver cancer cell lines.** (A) 18S rRNA is equally expressed between the different liver cancer cell lines. Real Time PCR experiments were performed using 18S rRNA as reference gene. An analysis of the Ct values of 18S rRNA revealed minor fluctuations confirming the suitability of 18S rRNA as reference gene. Results are shown as mean +/- SE for 3 (HCC-M and HuH6) to 4 independent experiments. (B) TGF-beta treatment of HCC cell lines resulted in no or negligible changes of expression of 18S rRNA in liver cancer cell lines. The diagram shows the mean deviation (ΔCt) of 18S rRNA Ct values from control and TGF-beta treated samples (24 h) of the same cell line. Results are presented as the mean +/- SE of 2-3 independent experiments.
(TIF)

Figure S2 **Densitometric analysis (ImageJ software) of Western Blots in Figure 1 and corresponding repetitive experiments.** Results are shown as mean +/- SE of the indicated numbers of independent experiments.
(TIF)

Figure S3 **Densitometric analysis (ImageJ software) of Western Blots presented in Figure 2 and corresponding repetitive experiments.** Results are shown as mean +/- SE of the indicated numbers of independent experiments.
(TIF)

Figure S4 **Densitometric analysis (ImageJ software) of Western Blots presented in Figure 3 and corresponding repetitive experiments.** Results are shown as mean +/- SE of the indicated numbers of independent experiments.
(TIF)

Figure S5 **Densitometric Analysis (ImageJ software) of Western Blots in Figure 5 and corresponding repetitive experiments.** Results are shown as mean +/- SE of the indicated numbers of independent experiments.
(TIF)

Figure S6 **Densitometric analysis ((ImageJ software) of Western Blots in Figure 6 and corresponding repetitive experiments.** Results are shown as mean +/- SE of the indicated numbers of experiments.
(TIF)
