# Supplementary figures and images for: Correction: Comparative Analysis of TGF-β/Smad Signaling Dependent Cytostasis in Human Hepatocellular Carcinoma Cell Lines
Source: PLoS One. 2014 May 8;9(5):e95952. doi: 10.1371/journal.pone.0095952 (PMC4014483; doi:10.1371/journal.pone.0095952)

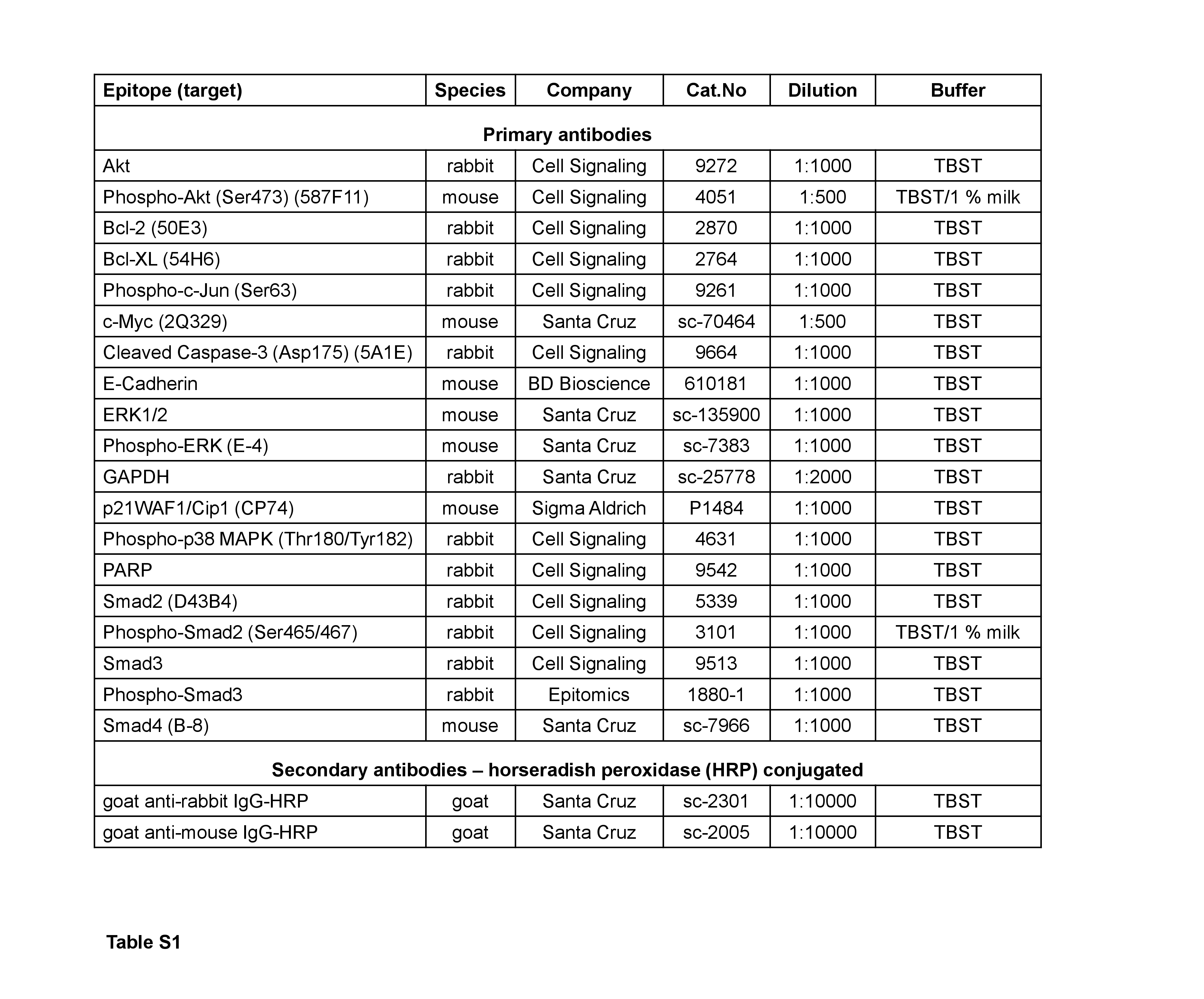

Supplement: Table S1 — Primary and secondary antibodies used for immunoblot analysis. Antibodies were obtained from Cell Signaling (Danvers, MA, USA), Santa Cruz Biotechnology (Santa Cruz, California, USA.), Sigma Aldrich (St. Louis, Missouri, USA), BD Bioscience (Heidelberg, Germany) or Epitomics (Burlingame, California, USA). After blocking the membrane with 5 % non fat milk powder in TBST, the membrane was incubated with the first antibody over night at 4 °C applying careful agitation. After removal of excessive antibodies, the membrane was incubated with the second antibody in TBST for 3-4 h at room temperature. (TIF) [file pone.0095952.s001.tif]

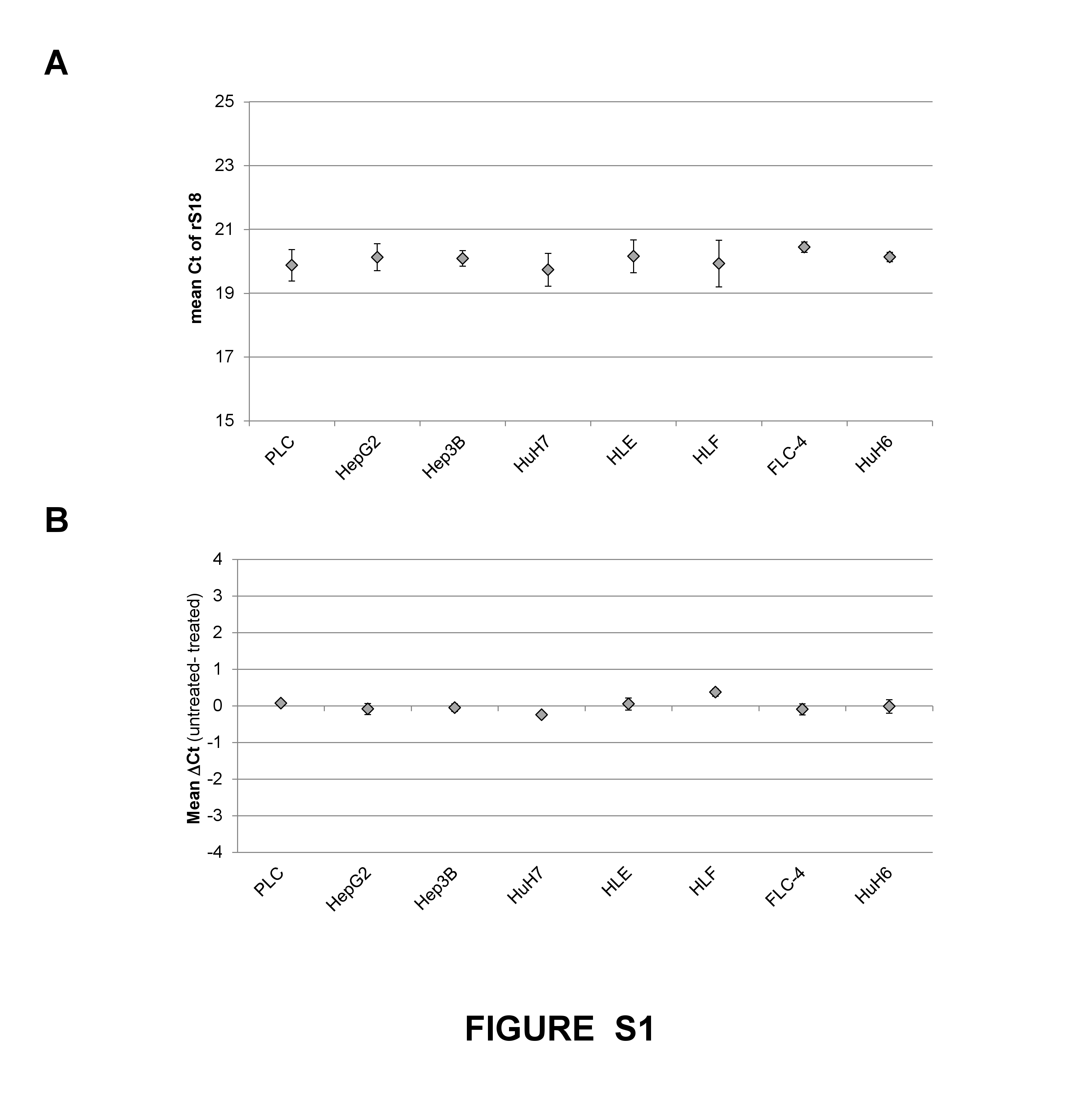

Supplement: Figure S1 — 18S rRNA is a suitable reference gene for real time PCR analysis of the used liver cancer cell lines. (A) 18S rRNA is equally expressed between the different liver cancer cell lines. Real Time PCR experiments were performed using 18S rRNA as reference gene. An analysis of the Ct values of 18S rRNA revealed minor fluctuations confirming the suitability of 18S rRNA as reference gene. Results are shown as mean +/- SE for 3 (HCC-M and HuH6) to 4 independent experiments. (B) TGF-beta treatment of HCC cell lines resulted in no or negligible changes of expression of 18S rRNA in liver cancer cell lines. The diagram shows the mean deviation (ΔCt) of 18S rRNA Ct values from control and TGF-beta treated samples (24 h) of the same cell line. Results are presented as the mean +/- SE of 2-3 independent experiments. (TIF) [file pone.0095952.s003.tif]

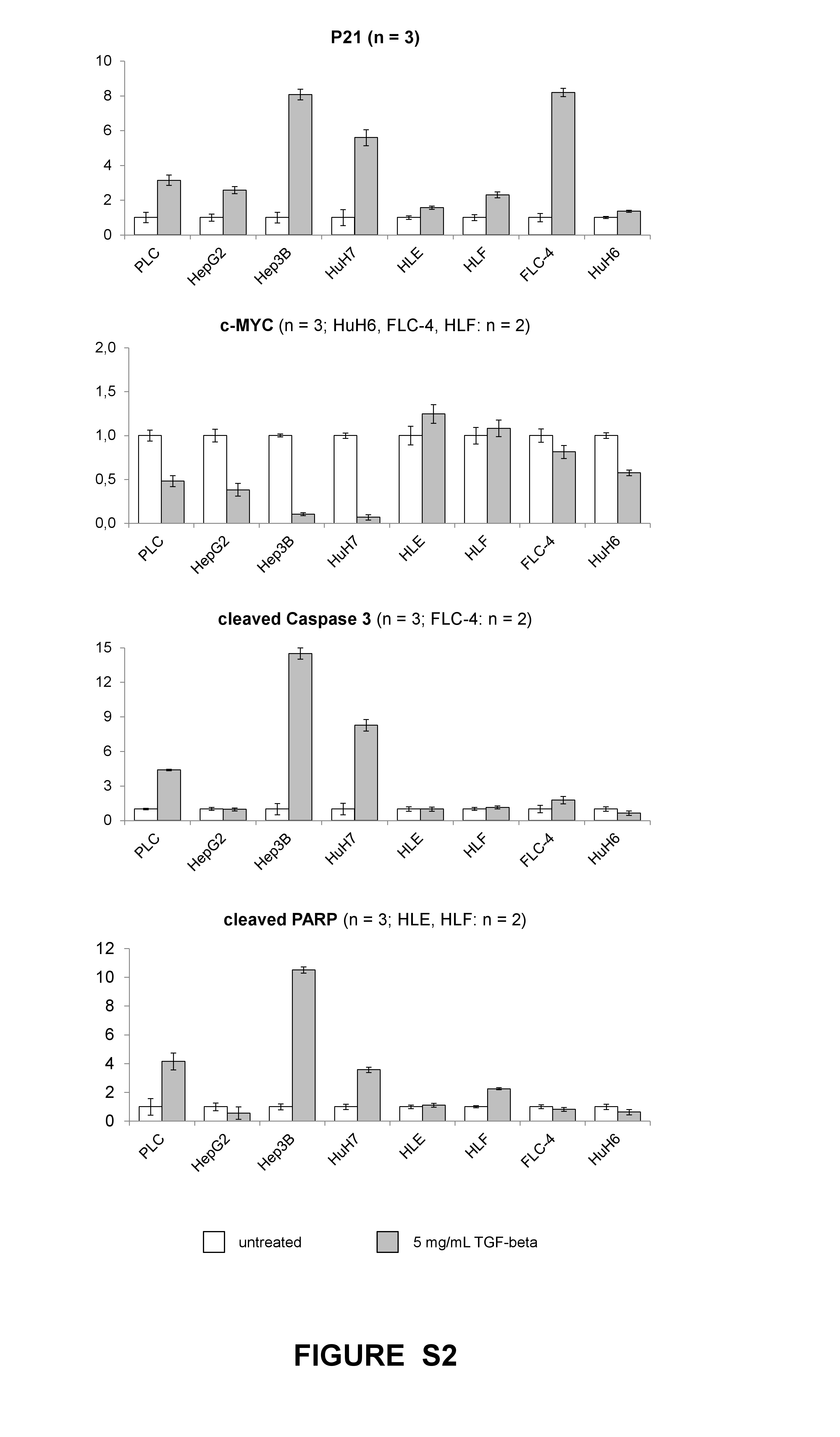

Supplement: Figure S2 — Densitometric analysis (ImageJ software) of Western Blots in Figure 1 and corresponding repetitive experiments. Results are shown as mean +/- SE of the indicated numbers of independent experiments. (TIF) [file pone.0095952.s004.tif]

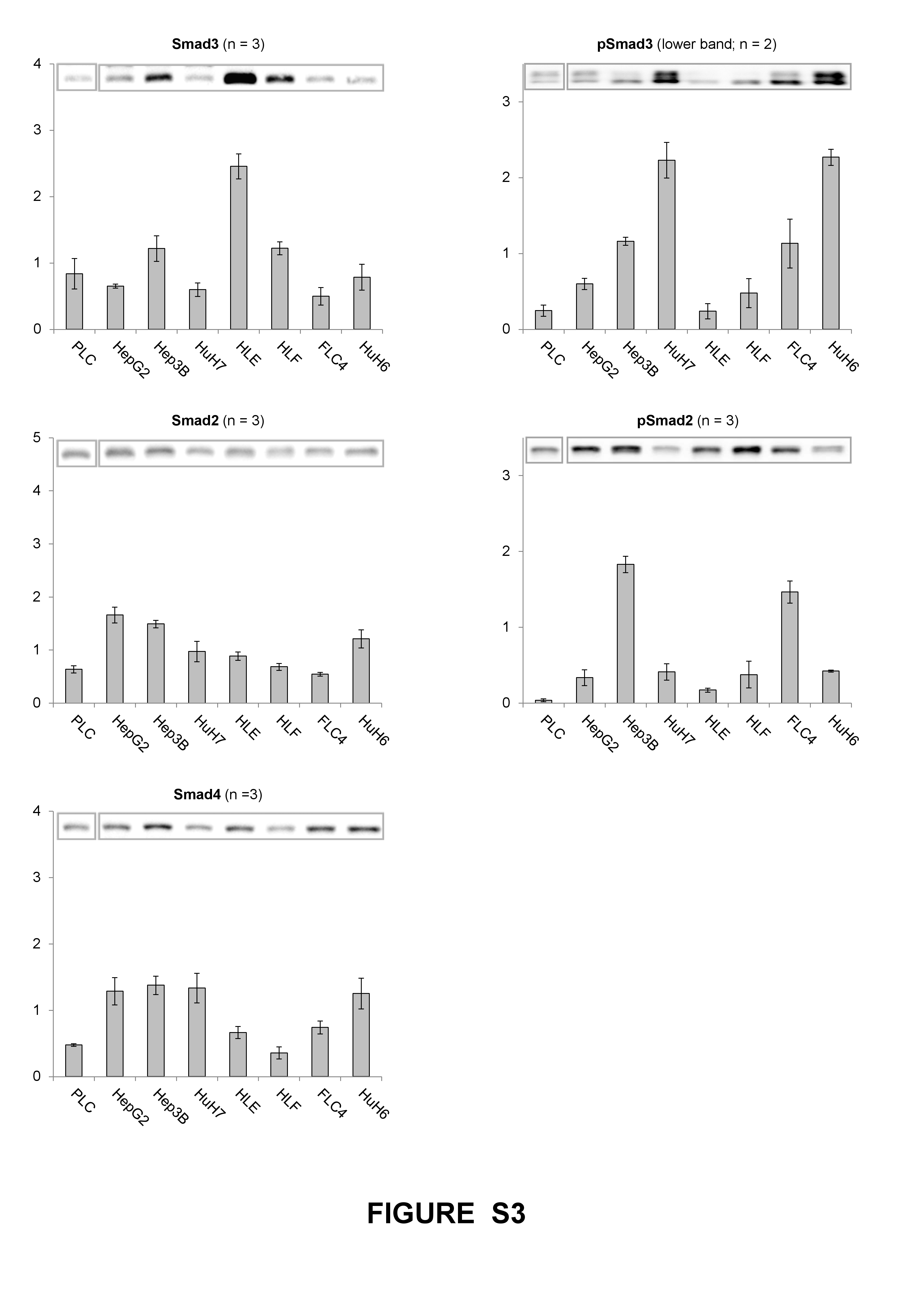

Supplement: Figure S3 — Densitometric analysis (ImageJ software) of Western Blots presented in Figure 2 and corresponding repetitive experiments. Results are shown as mean +/- SE of the indicated numbers of independent experiments. (TIF) [file pone.0095952.s005.tif]

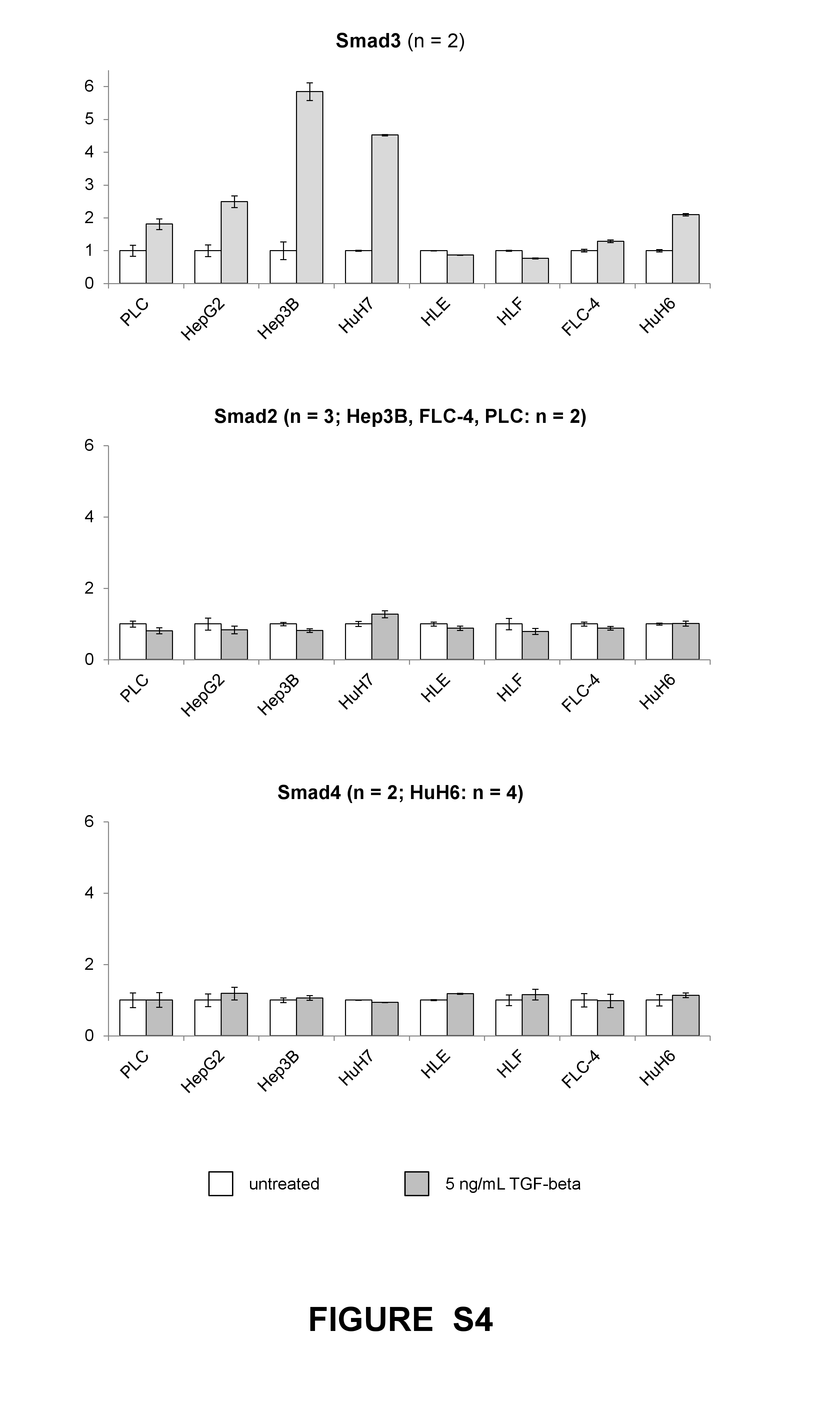

Supplement: Figure S4 — Densitometric analysis (ImageJ software) of Western Blots presented in Figure 3 and corresponding repetitive experiments. Results are shown as mean +/- SE of the indicated numbers of independent experiments. (TIF) [file pone.0095952.s006.tif]

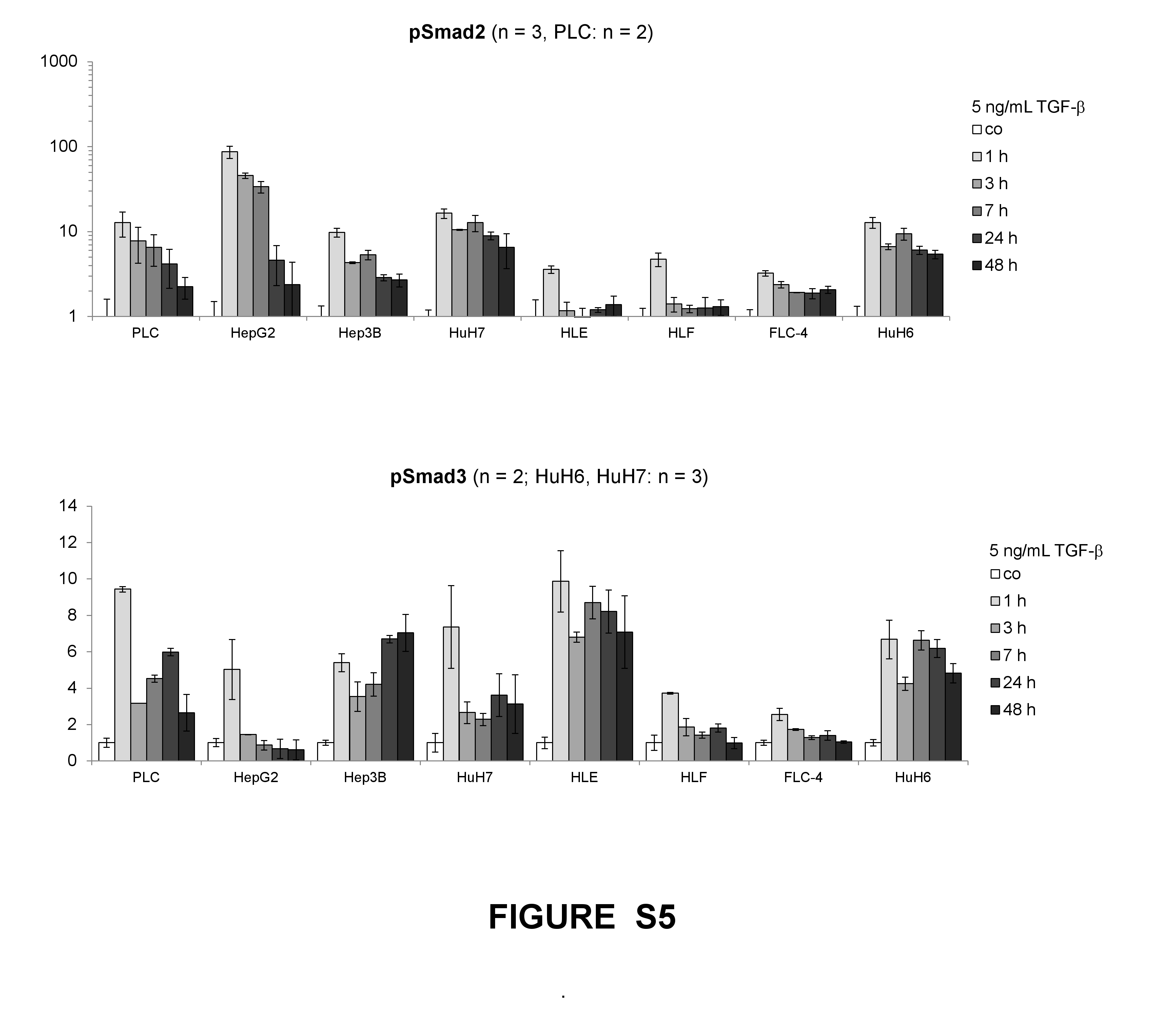

Supplement: Figure S5 — Densitometric Analysis (ImageJ software) of Western Blots in Figure 5 and corresponding repetitive experiments. Results are shown as mean +/- SE of the indicated numbers of independent experiments. (TIF) [file pone.0095952.s007.tif]

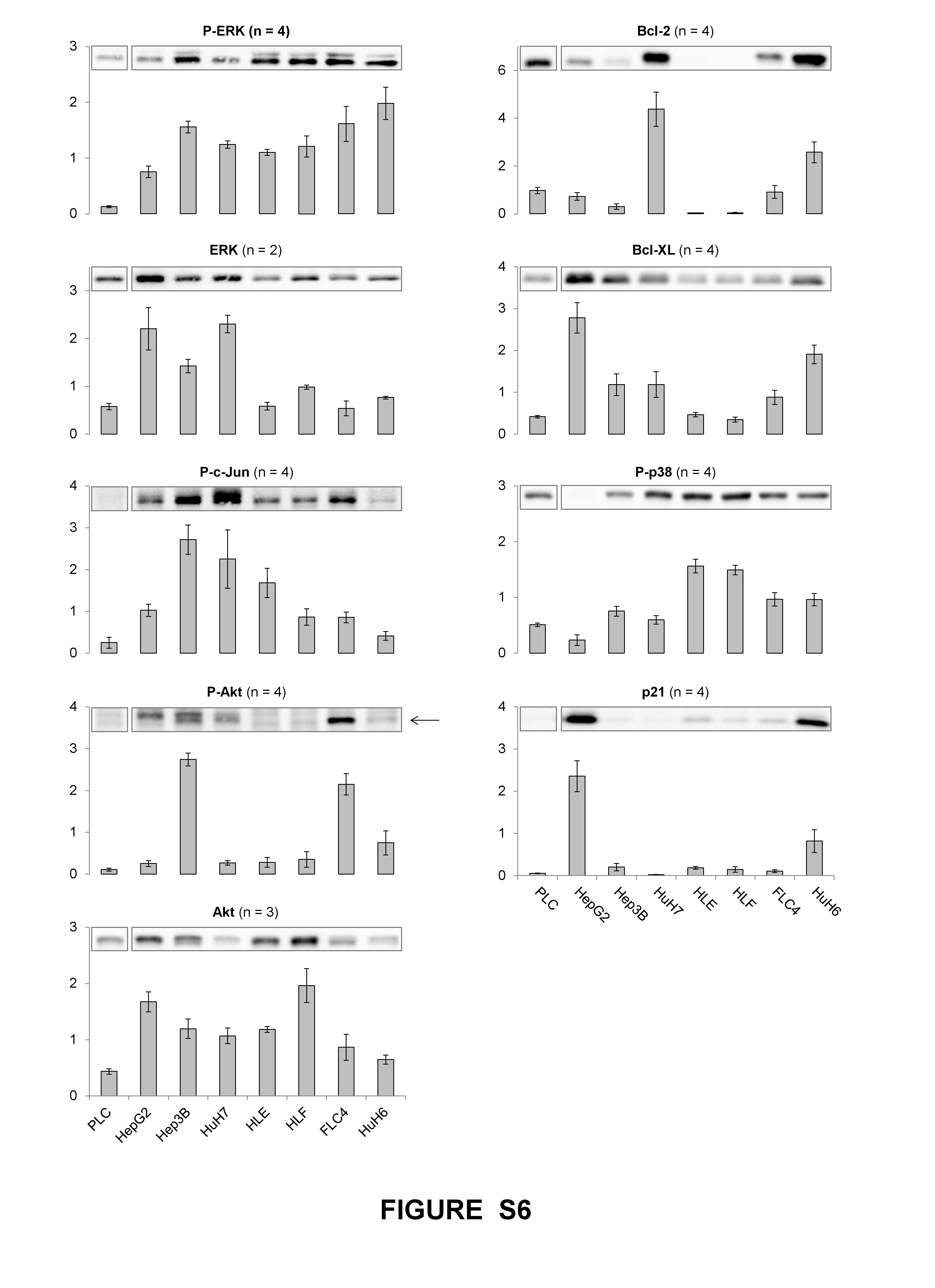

Supplement: Figure S6 — Densitometric analysis ((ImageJ software) of Western Blots in Figure 6 and corresponding repetitive experiments. Results are shown as mean +/- SE of the indicated numbers of experiments. (TIF) [file pone.0095952.s008.tif]
