# Supplementary material for: Correction: Comparative Analysis of TGF-β/Smad Signaling Dependent Cytostasis in Human Hepatocellular Carcinoma Cell Lines
Source: PLoS One. 2014 May 8;9(5):e95952. doi: 10.1371/journal.pone.0095952 (PMC4014483; doi:10.1371/journal.pone.0095952)
Supplement: Table S2 — Origin of the cell line, the original patient characteristics (age, sex, tumour stage), cell lines passages. (DOCX) [file pone.0095952.s002.docx]

|  | HCC-M | PLC | HCC-T | HepG2 | Hep3B | HuH7 | HLE | HLF | FLC-4 | HuH6 |
| --- | --- | --- | --- | --- | --- | --- | --- | --- | --- | --- |
| **Patients data** |  |  |  |  |  |  |  |  |  |  |
| Gender | m | m | m | m | m | m | m | m | m | m |
| Age | 34 | 24 | 69 | 15 | 8 | 57 | 68 | 68 | 51 | 1 |
| Tumor identity | hepatoma | hepatoma | HCC | hepatoma | hepatoma | hepatoma | hepatoma | hepatoma | HCC | Hepato  blastoma |
| Tumor stage  (Edmondson) | II | III | II |  |  |  |  |  | III |  |
| race | japanese | black | japanese | caucasian | black | japanese |  |  | japanese | japanese |
|  | | | | | | | | | | |
| **Cell line data** |  |  |  |  |  |  |  |  |  |  |
| Cell line dif-ferentiation | epithelial like | well | epithelial like | well | well | well | undifferentiated | undifferentiated | well | Epithelial like |
| E-Cadherin  expression | Not de-tectable | high | Not de-tectable | high | high | high | Very low | Not de-tectable | low | High |
| Passage when obtained | N/A | 8 | N/A | 7 | 5 | 11 | 8 | 8 | 3 | 32 |
| Passage number for experiments | 3-12 after thawing | | | | | | | | | |

**Table S2**
